# *MEG3* long noncoding RNA regulates the TGF-β pathway genes through formation of RNA–DNA triplex structures

**DOI:** 10.1038/ncomms8743

**Published:** 2015-07-24

**Authors:** Tanmoy Mondal, Santhilal Subhash, Roshan Vaid, Stefan Enroth, Sireesha Uday, Björn Reinius, Sanhita Mitra, Arif Mohammed, Alva Rani James, Emily Hoberg, Aristidis Moustakas, Ulf Gyllensten, Steven J.M. Jones, Claes M Gustafsson, Andrew H Sims, Fredrik Westerlund, Eduardo Gorab, Chandrasekhar Kanduri

**Affiliations:** 1 Department of Medical Genetics, Institute of Biomedicine, The Sahlgrenska Academy, University of Gothenburg, SE-40530 Gothenburg, Sweden; 2 Department of Immunology, Genetics and Pathology, Biomedical Center, SciLifeLab Uppsala, Uppsala University, SE-75108 Uppsala, Sweden; 3 Department of Medical Biochemistry and Cell Biology, University of Gothenburg, PO Box 440, SE-405 30 Gothenburg, Sweden; 4 Department of Medical Biochemistry and Microbiology, Science for Life Laboratory, Uppsala University, PO Box 582, SE-751 23 Uppsala, Sweden; 5 Ludwig Institute for Cancer Research, Science for Life Laboratory, Uppsala University, PO Box 595, SE-751 24 Uppsala, Sweden; 6 Genome Sciences Centre, British Columbia Cancer Agency, Vancouver, British Columbia BC V5Z 4S6, Canada; 7 Applied Bioinformatics of Cancer, University of Edinburgh Cancer Research UK Centre, Edinburgh EH4 2XR, UK; 8 Department of Chemical and Biological Engineering, Chalmers University of Technology, Gothenburg 412 96, Sweden; 9 Departamento de Genética e Biologia Evolutiva, Instituto de Biociências, Universidade de São Paulo, São Paulo CEP:05508-090, Brazil

## Abstract

Long noncoding RNAs (lncRNAs) regulate gene expression by association with chromatin, but how they target chromatin remains poorly understood. We have used chromatin RNA immunoprecipitation-coupled high-throughput sequencing to identify 276 lncRNAs enriched in repressive chromatin from breast cancer cells. Using one of the chromatin-interacting lncRNAs, *MEG3*, we explore the mechanisms by which lncRNAs target chromatin. Here we show that *MEG3* and *EZH2* share common target genes, including the TGF-β pathway genes. Genome-wide mapping of *MEG3* binding sites reveals that *MEG3* modulates the activity of TGF-β genes by binding to distal regulatory elements. *MEG3* binding sites have GA-rich sequences, which guide *MEG3* to the chromatin through RNA–DNA triplex formation. We have found that RNA–DNA triplex structures are widespread and are present over the *MEG3* binding sites associated with the TGF-β pathway genes. Our findings suggest that RNA–DNA triplex formation could be a general characteristic of target gene recognition by the chromatin-interacting lncRNAs.

## Abstract (web summary)

Long noncoding RNAs (lncRNAs) regulate gene expression by association with chromatin. Here, the authors show that lncRNA *MEG3* regulates the TGF-β pathway by bridging the interactions between polycomb repressive complex 2 and the distal regulatory elements of the TGF-β pathway genes via formation of RNA–DNA triplexes.

## Graphical abstract

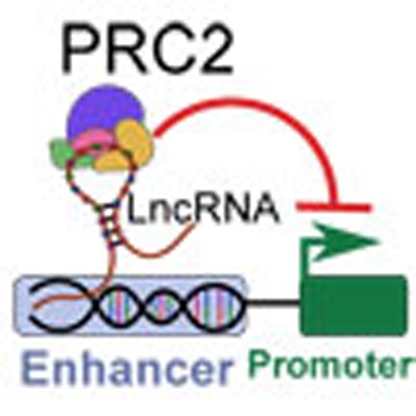

Long noncoding RNAs (lncRNAs) have emerged as key regulators of important biological processes implicated in development and differentiation^1^,^2^,^3^,^4^,^5^,^6^. Studies on the mode of action of lncRNAs have revealed that a subset of lncRNAs regulate gene expression in *cis* and *trans* by interacting with chromatin and recruiting chromatin modifiers^7^,^8^,^9^,^10^,^11^,^12^. Most studies to date have focused on identification of the RNA-interacting protein partners involved in gene activation or gene silencing^13^,^14^,^15^,^16^, and less attention has been paid in understanding how lncRNAs specifically target genes. Nevertheless, some recent investigations have provided insights into *Xist* targeting and its spreading along the inactive X chromosome (Xi)^17^,^18^. These studies did not predict any consensus binding sites by which *Xist* RNA is initially recruited before spreading along the Xi, but it has been proposed that the three-dimensional chromosomal conformation may play an important role in *Xist* spreading. On the other hand, chromatin-binding maps of *HOTAIR* and *Drosophila roX2* lncRNAs revealed that GA-rich sequences are the preferred binding motif, indicating that GA-rich sequences may help these RNAs to target the chromatin^19^. Identification of the lncRNAs that are associated with chromatin and exploration of the mechanistic aspects of the chromatin targeting of lncRNAs will help us to understand the molecular intricacies underlying lncRNA-dependent gene expression at the transcriptional level.

Active and inactive epigenetic modifications of the chromatin can regulate gene expression at the transcriptional level. When chromatin is enriched with repressive histone marks such as H3K27me3 and H3K9me3, it negatively regulates transcription^20^. The H3K27me3 histone modification is mediated by polycomb repressive complex 2 (PRC2). EZH2, EED and SUZ12 are the three major components of the PRC2 complex, where EZH2 is the catalytic subunit and EED is known to help in the propagation of H3K27me3 marks by allosteric activation of PRC2 (refs 21, 22). In *Drosophila*, the recruitment of PRC2 to the chromatin is mediated by specific sequences known as polycomb response elements. However, in mammals, it is not clear how sequence-specific recruitment of the PRC2 occurs across the genome. Recent evidence of a strong association between lncRNA and the PRC2 complex raise the possibility that lncRNAs may act as guiding molecules for PRC2 to target the chromatin^23^.

Several previous studies have focused on the identification of the polycomb-interacting lncRNAs either by using the RNA immunoprecipitation (RIP) technique or the photoactivatable ribonucleside-enhanced crosslinking and immunoprecipitation technique^24^,^25^,^26^,^27^. Although these studies have identified several PRC2-interacting lncRNAs, it remains unclear whether these lncRNAs are targeted to the chromatin.

Hence we sought to identify the repressive chromatin-associated lncRNAs on a global scale and also characterize the mechanisms by which these lncRNAs are targeted to the chromatin. Here we have characterized the repressive chromatin-associated lncRNAs on a genome-wide scale by performing chromatin RIP followed by high-throughput sequencing (ChRIP-seq) using antibodies to H3K27me3 and EZH2 in BT-549 cells. We identified 276 lncRNAs that are enriched in repressive chromatin. By using one of the chromatin-interacting lncRNAs (*MEG3*) as a model system, we explored the mechanisms by which it recognizes target genes. Consistent with ChRIP-seq data, *MEG3* interacts with the PRC2 complex. Through loss-of-function experiments of *MEG3* and *EZH2*, we found that *MEG3* in cooperation with PRC2 regulates a common set of genes, including those of the transforming growth factor-β (TGF-β) pathway. Using a modified chromatin oligo affinity precipitation (ChOP) method, we fine-mapped genome-wide chromatin-binding sites for *MEG3* RNA, revealing some of the TGF-β pathway genes as direct targets. *MEG3* binding sites showed enrichment in GA-rich sequences and we found that these GA-rich sequences guide *MEG3* RNA to its target genes through formation of RNA–DNA triplex structures. Our data demonstrate that RNA–DNA triplex structures are widespread *in vivo*, and are also present in the vicinity of the TGF-β pathway genes. Taken together, these results suggest that RNA–DNA triplex formation may be a general mechanism for target gene recognition by lncRNAs.

## Results

### Characterization of repressive chromatin-enriched lncRNAs

**Figure 1:**
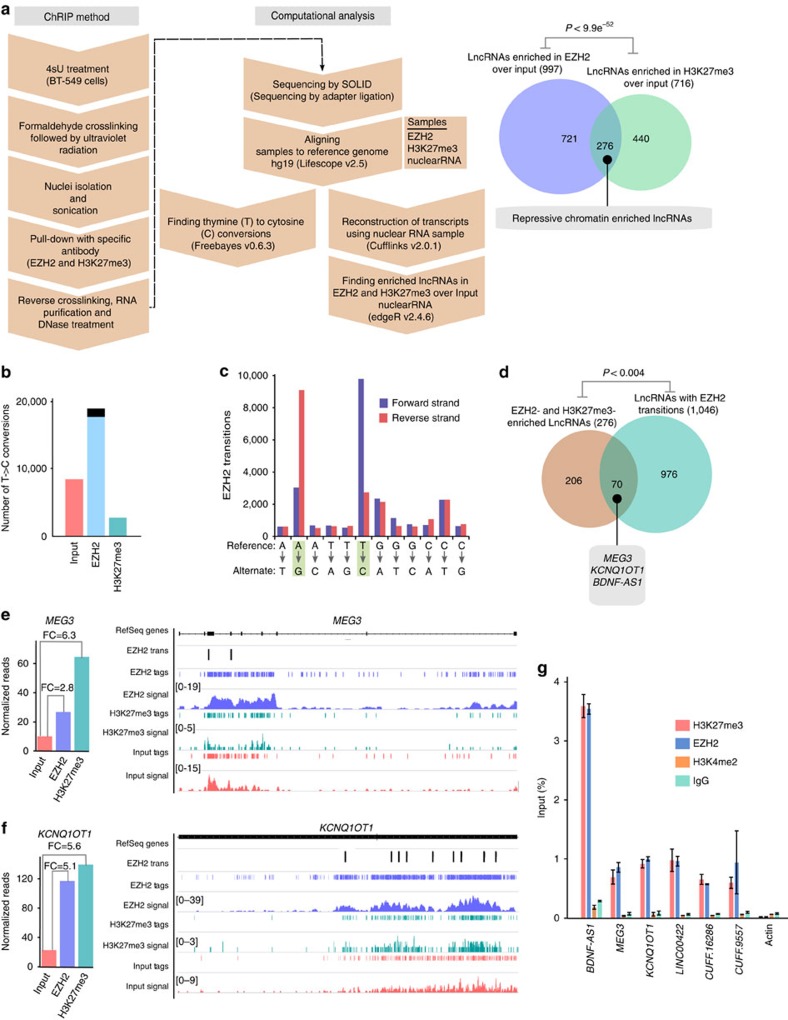
Identification of repressive chromatin-associated lncRNAs using ChRIP-seq. (**a**) The ChRIP-seq analysis pipeline used to identify lncRNAs enriched in repressive chromatin. The pie chart shows 276 lncRNAs enriched in both EZH2 and H3K27me3 ChRIP-seq samples compared with the nuclear RNA (input). The *P* value was obtained by performing a hypergeometric test using all the lncRNAs in our analysis. (**b**) Bar diagram showing the distribution of T-to-C transitions (indicative of putative RNA–protein contact sites) in input (8,361), EZH2 (18,905) and H3K27me3 (2,651) ChRIP-seq data. Black in the EZH2 bar indicates the number of T-to-C transitions (1,253) that are either present in input or H3K27me3 samples, and blue indicates EZH2-specific T-to-C transitions (17,652). The EZH2-specific T-to-C transitions (17,652) were used to associate with lncRNAs. (**c**) All the possible conversions present in the EZH2 ChRIP-seq sample. T-to-C conversion and the reverse-strand A-to-G conversions were predominant among all the possible conversion events. (**d**) LncRNAs (1,046; annotated and non-annotated) harbour EZH2-specific (17,652) T-to-C conversion site. Seventy repressive chromatin-enriched lncRNAs (out of 276) carry T-to-C transitions, including known PRC2-interacting lncRNAs such as *MEG3*, *KCNQ1OT1* and *BDNF-AS1.* The *P* value was obtained by performing a hypergeometric test using all the lncRNAs considered in our analysis. (**e,f**) The distribution of the sequencing reads on *MEG3* and *KCNQ1OT1* transcripts from H3K27me3, EZH2-enriched chromatin fractions and input RNA samples. The tags represent the read distribution and the signal represents the intensity of reads over *MEG3* and *KCNQ1OT1* transcripts. Locations of T-to-C transitions over the exons are depicted below the physical maps. The left panel depicts the RPKM (Reads per kilobase per million) for *MEG3* and *KCNQ1OT1* in H3K27me3, EZH2 ChRIP RNA and input RNA samples. The fold enrichment (FC) in H3K27me3 and EZH2 ChRIP RNA compared with input is indicated. (**g**) ChRIP validation: RT–qPCR data showing the enrichment of the selected annotated and non-annotated lncRNAs in the EZH2 and H3K27me3 ChRIP pull-downs compared with input. We did not observe any enrichment of these lncRNAs in the H3K4me2 (active chromatin marks) and immunoglobulin G (IgG; nonspecific antibody) ChRIP pull-downs. Actin was used as a negative control. Data represent the mean±s.d. of three independent biological experiments.

Previously, we have used ChRIP to verify the chromatin association of the mouse *Kcnq1ot1* antisense lncRNA^10^. Here we used a modified ChRIP protocol in combination with photoactivatable ribonucleside-enhanced crosslinking followed by high-throughput sequencing (ChRIP-seq) to identify lncRNAs that are associated with repressive chromatin on a global scale (Fig. 1a). In brief, we incubated BT-549 cells overnight (14–16 h) with 4-thiouridine (4sU), followed by a 40-min incubation with actinomycin D (ActD). ActD-treated BT-549 cells were crosslinked with formaldehyde, followed by ultraviolet irradiation. 4sU-incorporated RNA can be crosslinked with proteins *in vivo* by ultraviolet irradiation. Crosslinking with formaldehyde ensures stabilization of the chromatin-interacting lncRNAs to the chromatin. Incubation of BT-549 cells with ActD before crosslinking blocks transcription, which in turn prevents the co-transcriptional crosslinking of lncRNAs to the chromatin. The efficacy of the transcriptional arrest by ActD was tested using short half-life mRNA *C-MYC* as described previously (Supplementary Fig. 1a)^28^. Chromatin was prepared from the formaldehyde and ultraviolet -crosslinked BT-549 cells, and was subjected to immunoprecipitation using antibodies to H3K27me3 and EZH2. The specificity of the immunopurified chromatin was tested by quantitative PCR (qPCR) with positive and negative controls (Supplementary Fig. 1b). After reversal of crosslinking, RNA was isolated from the immunoprecipitated chromatin. Isolated RNA was extensively treated with DNase I to remove all traces of DNA, and verified again by qPCR (Supplementary Fig. 1b). The DNase I-treated anti-H3K27me3 and anti-EZH2 purified RNAs along with nuclear input RNA were subjected to high-throughput sequencing. The reconstruction of the nuclear RNA using Cufflinks revealed previously annotated lncRNAs and also non-annotated transcripts. Coding potential analysis of the non-annotated transcripts found that they had lower coding probabilities, suggesting that they are noncoding RNAs (Supplementary Fig. 1c). We looked for enrichment of the annotated and non-annotated transcripts in H3K27me3 and EZH2 ChRIP-purified RNA fractions over nuclear input (Supplementary Data 1 and 2). We considered lncRNAs in our ChRIP data set to be ‘repressive chromatin enriched' only if they were enriched (minimum twofold) in both H3K27me3 and EZH2 ChRIP-purified RNA fractions compared with the nuclear input. We found a significant overlap (276 lncRNAs, *P*<9.9e^−52^, hypergeometric distribution) between H3K27me3 and EZH2 ChRIP pull-downs (Fig. 1a). The list of 276 lncRNAs enriched in repressive chromatin comprises both annotated and non-annotated transcripts (Supplementary Data 3). The 4sU incorporation provided us with an additional advantage in our RNA sequencing data, as the possible protein interaction sites on RNA lead to ultraviolet-induced T-to-C transitions. The T-to-C conversions at the putative RNA–protein contact sites in ChRIP RNA sequencing samples were considered only if the minimum sequencing read depth over the conversions was ≥2 (read depth indicates total number of sequencing reads covered per transition)^29^. Using this criterion, we observed that T-to-C conversion was overrepresented in the EZH2 ChRIP RNA fraction in comparison with both the H3K27me3 ChRIP RNA and input RNA (Fig. 1b). We found that the overrepresentation of T-to-C conversion in EZH2 ChRIP data was not by chance, as the other nucleotide conversions were detected at background level in the EZH2 ChRIP data compared with T to C (A to G also represents T-to-C conversion in the reverse strand of RNA sequencing data; Fig. 1c). We identified 17,652 T-to-C conversions that were present only in EZH2 ChRIP data but not in H3K27me3 and input RNA data. These T-to-C conversions were then mapped to annotated and non-annotated transcripts, reconstructed from nuclear RNA input, revealing 1,046 lncRNAs with putative RNA–protein contact sites. We found a significant overlap between these lncRNAs and the lncRNAs that were enriched in EZH2 ChRIP and in repressive chromatin (enriched in both H3K27me3 and EZH2 ChRIPs) (Fig. 1d, Supplementary Fig. 1d and Supplementary Data 4). The presence of EZH2 ChRIP-specific T-to-C conversion sites over the repressive chromatin-associated lncRNAs indicates that they are either putative EZH2 contact sites or EZH2-associated protein contact sites over the lncRNAs. Interestingly, the 70 repressive chromatin-associated lncRNAs with T-to-C conversions contain several annotated and non-annotated (both intergenic and intronic) lncRNAs (Supplementary Data 4 and Supplementary Fig. 2a,b), including three known PRC2-interacting lncRNAs: *KCNQ1OT1*, *MEG3* and *BDNF-AS1* (Fig. 1e,f and Supplementary Data 4). Mouse orthologues *Kcnq1ot1* and *Gtl2* have been shown to interact with PRC2, and moreover *Kcnq1ot1* has also been shown to be enriched in the mouse placental chromatin fraction^10^,^24^,^30^. We validated the repressive chromatin enrichment of some of the annotated lncRNAs (*BDNF-AS1*, *MEG3*, *KCNQ1OT1* and *LINC00422*) and non-annotated lncRNAs (intergenic *CUFF.16286* and intronic *CUFF.9557*) using qPCR assay on ActD-treated and -untreated ChRIP materials (Fig. 1g and Supplementary Fig. 2c).

### Mapping PRC2-interacting region of *MEG3* lncRNA

**Figure 2:**
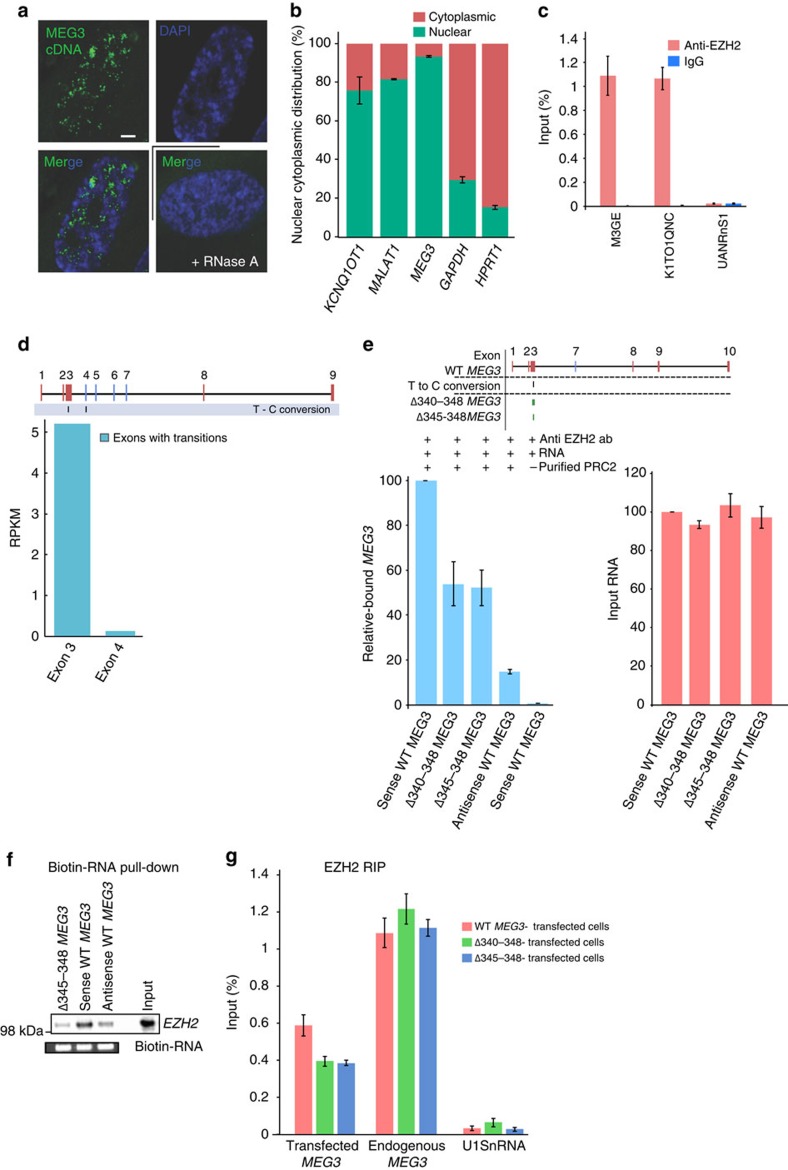
Molecular characterization of *MEG3* and PRC2 interaction. (**a**) RNA-fluorescence *in situ* hybridization showing the distribution of the *MEG3* signal (green) in the nucleus (blue, stained with 4,6-diamidino-2-phenylindole). An RNase A-treated sample was used as a negative control. Scale bar, 1 μm. (**b**) RT–qPCR data showing the distribution of lncRNAs and protein-coding RNAs in the nuclear and cytoplasmic fractions (±s.d., *n*=3). (**c**) RT–qPCR analysis of *MEG3*, *KCNQ1OT1* and *U1SnRNA* in EZH2 RIP-purified RNA from BT-549 cells. *U1SnRNA* served as negative control. The enrichment is plotted as percentage of input (±s.d., *n*=3). (**d**) Physical map of the *MEG3* containing numbered exons showing two T-to-C transitions. The exons in red are constitutively expressed and blue are alternatively spliced exons. First conversion is part of exon 3 showing higher expression, whereas the second conversion is part of exon 4 showing low expression in the nuclear RNA sequencing. (**e**) *In vitro* interaction of *MEG3* and PRC2. The schematic indicates the exons of the WT *MEG3* clone. Left: RT–qPCR showing enrichment of sense WT *MEG3* and *MEG3* carrying deletions (Δ340-348 or Δ345-348 *MEG3*) in *in vitro* RNA binding assays. Reaction with antisense WT *MEG3* or without purified PRC2 served as negative controls. The binding efficiency of *MEG3* deletions were presented relative to WT *MEG3* (±s.d., *n*=3). Right: RT–qPCR showing the quantification of input RNAs. (**f**) Upper panel: western blot showing EZH2 levels after pull-down with biotinylated sense WT *MEG3*, antisense WT *MEG3*, and Δ345-348 *MEG3* RNAs incubated with nuclear extract. This is a representative data set from several experiments. Lower panel: agarose gel picture showing input biotin-RNA. (**g**) RT–qPCR result showing the relative enrichment of WT *MEG3,* Δ340-348 and Δ345-348 *MEG3* RNAs in the EZH2-RIP, performed after BT-549 cells were transfected with WT and mutant *MEG3* plasmids. Data were normalized to the input RNAs and plotted as percentage of input (±s.d., *n*=3). To distinguish the endogenous *MEG3* from the ectopically expressed *MEG3*, we designed RT–qPCRs primers, with one primer mapped to the transcribed portion of the vector and the other to *MEG3* RNA. Endogenous *MEG3* served as positive control and *U1SnRNA* as negative control.

Since *MEG3* lncRNA was identified as a repressive chromatin-associated RNA in the ChRIP analysis (Fig. 1d,e), and also as one of the chromatin-interacting RNAs in our previous study involving sucrose-fractionated chromatin from normal human fibroblasts (HF cells)^28^, we were interested in exploring plausible mechanisms by which *MEG3* lncRNA recognizes its target genes. Human *MEG3* is an lncRNA of ∼1,700 nucleotides with different isoforms generated by alternative splicing. Exons 1–3 and 8–9 are common to all isoforms, whereas exons 4–7 are present in different combinations^31^. *In situ* RNA hybridization and nuclear–cytoplasmic RNA fractionation experiments indicated that *MEG3* is located in the nuclear compartment (Fig. 2a,b). We checked for the interaction of *MEG3* lncRNA with PRC2 by RIP and found robust enrichment of *MEG3* in the PRC2-interacting RNA fraction (Fig. 2c), and its fold enrichment was more or less similar to the enrichment of *KCNQ1OT1* lncRNA (Fig. 2c), which was used as positive control for the RIP experiment.

We then wanted to fine-map the sequences of *MEG3* RNA that dictate interaction of PRC2 with *MEG3*. To this end, we looked for the status of the incorporated 4sU nucleotide conversions (T to C) in the EZH2 ChRIP-seq data, which indicates possible RNA–protein contact points. We detected two converted nucleotides at the 5′-end of the *MEG3* RNA in the EZH2 immunopurified RNA (Figs 1e and 2d), indicating that the conversions could be possible contact points for EZH2 or EZH2-associated protein contact sites. The first conversion was located in a constitutively expressed exon 3, whereas the second one was in an alternatively spliced exon 4 (ref. 31). We PCR-amplified full-length *MEG3* clones using nuclear RNA from BT-549 cells (hereon referred to as wild-type (WT) *MEG3*). It contains all the constitutively expressed exons (that is, exons 1–3 and 8–9) and an alternatively spliced exon 7 (out of the four alternatively spliced exons 4–7), but not exon 4. Moreover, we detected fewer reads in the nuclear RNA over the alternatively spliced exon with the second T-to-C conversion (Fig. 2d). Since we failed to detect the alternatively spliced exon 4 in several full-length amplified *MEG3* clones, we therefore focused on the first T-to-C conversion located in the constitutively expressed exon 3. We inserted two deletions of 4 bp (345–348) and 9 bp (340–348) (Fig. 2e), overlapping the first conversion site in exon 3, into a WT *MEG3* clone. We carried out an *in vitro* binding assay with WT and mutant (Δ340-348 *MEG3* and Δ345-348 *MEG3*) *MEG3* RNAs using purified PRC2 complex, comprising EZH2, EED and SUZ12 subunits (Fig. 2e and Supplementary Fig. 3a). An antisense version of the full-length *MEG3* (antisense WT *MEG3*) was used as a negative control. We found that the PRC2 binding was partially compromised in the mutant *MEG3* RNAs (Δ340-348 *MEG3* and Δ345-348 *MEG3*) compared with the WT *MEG3* (Fig. 2e). We also tested the binding of WT and Δ345-348 *MEG3* RNAs with PRC2 using the nuclear lysate from BT-549 cells, and found that the sense WT *MEG3*, but not the Δ345-348 *MEG3* RNA, efficiently bound to PRC2, as detected by EZH2 immunoblot (Fig. 2f). To further test the efficacy of the association of PRC2 with the WT and mutant *MEG3* RNAs *in vivo*, we transfected BT-549 cells with the plasmids expressing WT, Δ340-348 and Δ345-348 *MEG3* RNAs, and performed RIP. By using primer combinations that selectively amplify the transfected plasmid-derived *MEG3* RNAs, we found that Δ340-348 *MEG3* and Δ345-348 *MEG3* transcripts were less enriched than the WT-transfected *MEG3*, whereas endogenous *MEG3* was equally enriched in all pull-downs (Fig. 2g). Thus, by using 4sU conversion data along with *in vitro* and *in vivo* binding experiments, we had fine-mapped the probable contact points for PRC2 interaction in the human *MEG3* lncRNA.

### *MEG3*/*EZH2* interaction regulates the TGF-β pathway genes

**Figure 3:**
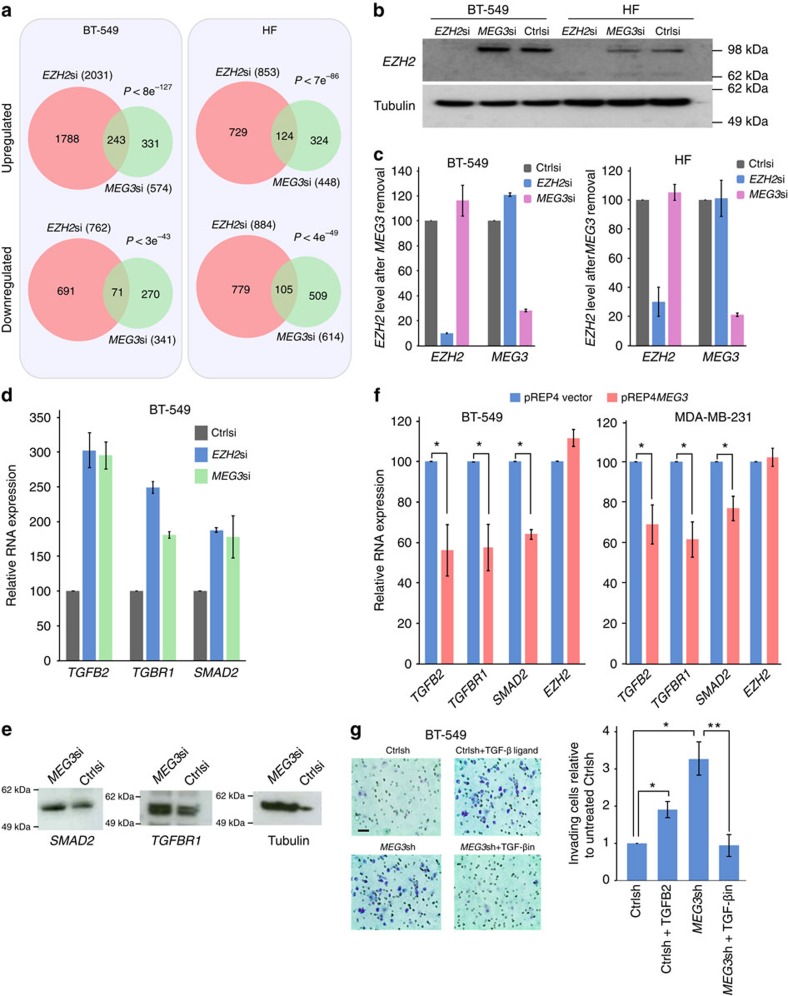
*MEG3*/*EZH2* functional interaction regulates TGF-β pathway genes. (**a–c**) *MEG3* and *EZH2* share common gene targets. (**a**) Venn diagram showing the number of genes deregulated after downregulation of *MEG3* and *EZH2* using siRNA in BT-549 and HF cells, and the degree of overlap between the *MEG3-* and EZH2-dependent genes. The *P* values were obtained by hypergeometric test using all protein-coding genes as a background. (**b**) EZH2 protein levels, as determined by western blotting, following EZH2 and *MEG3* downregulation in BT-549 and HF cells. Tubulin was used as a loading control. (**c**) RT–qPCR analysis of *EZH2* and *MEG3* mRNA expression in Ctrlsi, *EZH2*si and *MEG3*si transfected BT-549 and HF cells (±s.d., *n*=3). (**d**) RT–qPCR analysis of *TGFB2*, *TGFBR1* and *SMAD2* gene expression in Ctrlsi, *MEG3*si and *EZH2*si transfected BT-549 cells (±s.d., *n*=3). (**e**) Immunoblot showing SMAD2, TGFBR1 and tubulin protein levels following transfection of BT-549 cells with Ctrlsi and *MEG3*si. (**f**) Bar graph showing RT–qPCR analysis of *TGFB2*, *TGFBR1* and *SMAD2* mRNA levels after overexpression of *MEG3* (pREP4*MEG3*) in BT-549 and MDA-MB-231 cells. The levels in pREP4*MEG3* are presented relative to CtrlpREP4 (±s.d., *n*=3). *EZH2* was used as a control showing no change in expression after overexpression of *MEG3*. The *P* values were calculated using Student's *t*-test (two-tailed, two-sample unequal variance), *P<0.05. (**g**) Downregulation of *MEG3* influences the invasive property of BT-549 cells through regulation of the TGF-β pathway. Images showing Matrigel invasion of the BT-549 cells. The two images in the upper panel show invasion of BT-549 cells infected with Ctrlsh lentivirus, and Ctrlsh infection followed by incubation with TGF-β2 ligand (Ctrlsh-TGFB2). The images in the bottom panel show the cells infected with *MEG3*Sh and *MEG3*sh infection followed by incubation with TGF-β inhibitor (*MEG3*sh+*TGF-β*in). Scale bar, 10 μm. The bar graph shows quantification (±s.d., *n*=3) of the matrix invaded cells in *MEG3*sh relative to the Ctrlsh. The *P* values were calculated using Student's *t*-test (two-tailed, two-sample unequal variance), *P<0.05, **P<0.01.

To gain more insights into the functional significance of the interaction of PRC2 with the *MEG3* lncRNA, both *EZH2* and *MEG3* transcripts were downregulated in BT-549 and HF cells using small interfering RNA (siRNA), and gene expression profiles were measured using microarray. We observed a significant overlap among the deregulated genes between the *EZH2* and *MEG3* data sets from BT-549 and HF cells, indicating a functional association between the *MEG3* lncRNA and EZH2 (Fig. 3a, Supplementary Data 5 and Supplementary Data 6). *MEG3* downregulation did not interfere with *EZH2* RNA and protein levels, and similarly, *EZH2* downregulation did not affect *MEG3* transcript levels (Fig. 3b,c), suggesting that the overlap observed between the deregulated genes in the *EZH2* and *MEG3* data sets was not due to changes in *EZH2* levels upon *MEG3* downregulation or in *MEG3* levels upon *EZH2* downregulation. To rule out off-target effects of the *MEG3* siRNA, we used reverse transcription (RT)–qPCR to validate four target genes upon *MEG3* downregulation using an alternative siRNA against *MEG3* (Supplementary Fig. 3b,c).

Pathway analysis of the differentially expressed genes identified from microarray revealed that several pathways were affected in common after *MEG3* and *EZH2* removal (Table 1 and Supplementary Data 7). We also performed RNA sequencing of the *MEG3*/*EZH2* downregulated samples from an independent biological experiment, and found a significant overlap among differentially expressed genes from the RNA sequencing and microarray experiments (Supplementary Fig. 4a). Pathway analysis of the RNA sequencing samples also revealed the same pathways as those obtained using microarray experiments, further suggesting a functional interaction between *MEG3* and *EZH2* (Table 1 and Supplementary Data 7). In addition, similar pathways were obtained with the commonly deregulated genes from microarray and RNA sequencing experiments upon *MEG3* and *EZH2* downregulation (Supplementary Fig. 4b). Since the TGF-β pathway is one of the well-investigated signalling cascades in mammals among the affected pathways, we were interested in understanding the functional role of *MEG3* in the regulation of genes involved in the TGF-β pathway. We validated the differential expression of the key TGF-β pathway genes *TGFB2*, *TGFBR1* and *SMAD2* in BT-549 and HF cells after siRNA-mediated downregulation of *EZH2* and *MEG3* transcripts (Fig. 3d,e and Supplementary Fig. 4c). The overexpression of full-length *MEG3* and *EZH2* resulted in significant downregulation of the TGF-β pathway genes *TGFB2*, *TGFBR1* and *SMAD2* (Fig. 3f and Supplementary Fig. 4d). Activation of the TGF-β pathway target genes *ACTC1*, *CNN1* and *COL5A1*, with functional roles in cytoskeletal organization, was also observed upon downregulation of *EZH2* and *MEG3* by siRNA (Supplementary Fig. 5a). We further confirmed the TGF-β pathway-mediated regulation of these genes by treating BT-549 cells with TGF-β2 ligand for 24 h, and, as expected, we observed activation of expression of the *ACTC1*, *CNN1* and *COL5A1* genes (Supplementary Fig. 5b). We also observed an additive effect on the activation of these genes if the cells were treated with both *MEG3* siRNA and TGF-β ligand together (Supplementary Fig. 5c). Activation of these genes was not observed when the cells were treated with *MEG3* siRNA along with a TGF-β inhibitor (Supplementary Fig. 5c). These results together highlight how *MEG3* regulates its secondary gene targets through control of the primary TGF-β target genes.

Considering the functional role of the TGF-β pathway in the regulation of cell invasion^32^,^33^,^34^, we investigated whether the activated the TGF-β pathway members in the *MEG3*-downregulated cells enhance cell invasion. To this end, we performed a Matrigel cell invasion assay with the BT-549 cells transduced with lentiviral particles containing *MEG3* and control short hairpin RNAs (shRNAs). Compared with the control shRNA, the *MEG3* shRNA-transduced BT-549 cells showed activation of the TGF-β pathway genes (Supplementary Fig. 5d) and also showed a significant increase in their invasion through Matrigel (Fig. 3g). The increased invasion of the *MEG3* shRNA cells was reversed when the cells were treated with TGF-β inhibitor (Fig. 3g). We also observed an increase in the cell invasion of the control shRNA-expressing cells in the presence of TGF-β2 ligand (Fig. 3g). These results suggest that *MEG3* partly controls the cell invasion of BT-549 cells through regulation of the TGF-β pathway. When we overexpressed *MEG3* in MDA-MB-231 cells, we found that there was a significant decrease in the invasive capacity of the MDA-MB-231 cells, indicating that *MEG3* RNA suppresses cell invasion (Supplementary Fig. 5e).

Given the functional interaction between *MEG3* expression and *TGF-β* gene regulation in the breast cancer cell line BT-549, we extended our cell line analyses to the published clinical breast cancer data sets. We found that *MEG3* is expressed at significantly lower levels in invasive ductal carcinoma than in normal breast tissue (Supplementary Fig. 5f). We then integrated gene expression data from 17 published studies, representing 2,999 primary breast tumours, and found that *MEG3* had the lowest average expression and widest range of expression in the aggressive and difficult-to-treat basal molecular subtype (Supplementary Fig. 5g). Consistent with this observation, *MEG3* is expressed at lower levels in high-grade breast tumours (Supplementary Fig. 5h). We also observed that *TGFB2, TGFBR1* and *SMAD2* genes (Supplementary Fig. 5i) had greater expression in tumours with low *MEG3* expression, further supporting our *in vitro* cell culture results that *MEG3* negatively regulates the TGF-β pathway genes (Fig. 3).

### *MEG3* binds to distal regulatory elements of *TGF-β* genes

**Figure 4:**
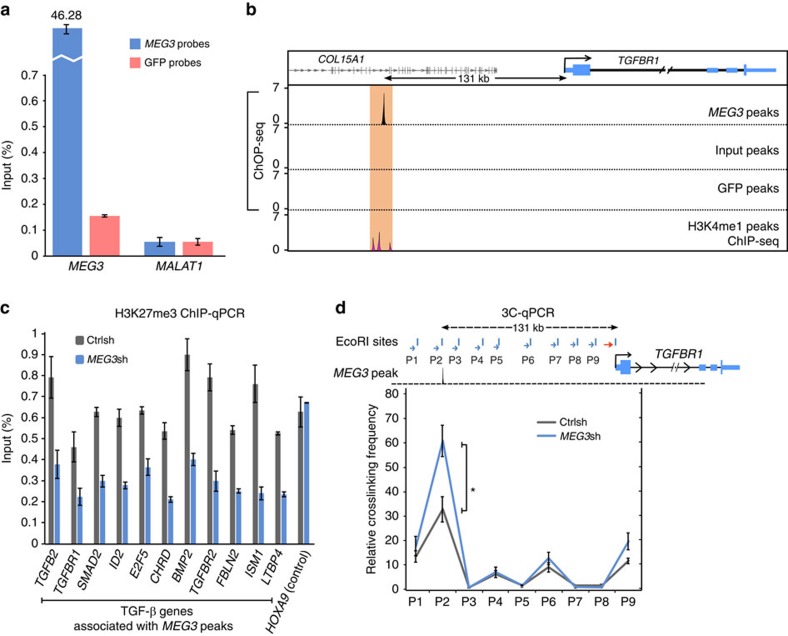
Genome-wide mapping of *MEG3* lncRNA binding sites. (**a**) RT–qPCR analysis showing specific enrichment (presented as percentage of input) of *MEG3* but not *MALAT1* RNA in the ChOP pull-down assay with *MEG3* antisense probes. The ChOP pull-down with GFP antisense oligo, used as a negative control, did not show any enrichment of *MEG3* and *MALAT1* RNAs. (**b**) Genomic tracks showing ChOP-seq (*MEG3*, GFP and input) and ChIP-seq (H3K4me1) intensities, visualized in log scale. The *MEG3* binding site is located upstream of the *TGFBR1* gene (falls within the intron of the *COL15A1* gene) and it overlaps with H3K4me1 peaks in BT-549 cells. (**c**) ChIP–qPCR showing enrichment of H3K27me3 chromatin marks, presented as percentage of input, over the *MEG3* peaks associated with the *TGF-β* genes in Ctrlsh and *MEG3*sh cells (±s.d., *n*=3). (**d**) Schematic outline of the *TGFBR1* gene showing *MEG3* peaks and the location of 3C primers (P1–P9), as indicated by arrows. EcoRI restriction sites are shown as blue vertical lines. Each error bar represents ±s.d. from three experiments. Looping events between the upstream *MEG3* binding site (corresponding to P2 primer) and the *TGFBR1* promoter detected by 3C–qPCR in Ctrlsh and *MEG3*sh cells. The *P* values were calculated using Student's *t*-test (two-tailed, two-sample unequal variance), *P<0.05.

We next wanted to address an important question: how does *MEG3* target the multiple TGF-β pathway genes in *trans*? For this purpose, we wanted to fine-map genome-wide *MEG3* binding sites using the ChOP method with minor modifications. We have previously used ChOP methodology to characterize the *Kcnq1ot1* lncRNA binding sites on mouse chromosome 7 (refs 10, 35). This method is conceptually equivalent to other methods currently used to fine-map RNA binding sites^16^,^17^,^19^,^36^. We used 15 biotin-labelled antisense DNA oligonucleotides (oligos) spanning across *MEG3* RNA (Supplementary Fig. 6a) to ensure robust capture of *MEG3* RNA-associated genomic loci with streptavidin beads. The ChOP pull-down using *MEG3* antisense oligos detected specific enrichment of the *MEG3* RNA, but not abundantly expressed nuclear-enriched *MALAT1* lncRNA, whereas pull-down with a biotin probe against green fluorescence protein (GFP) RNA (with no known target in the human genome), used as a negative control, detected neither *MEG3* nor *MALAT1*, highlighting the specificity of the ChOP pull-down assay (Fig. 4a). We then subjected the ChOP pull-down chromatin material with *MEG3* and control probes to high-throughput DNA sequencing. By considering the *MEG3*-enriched regions over input and nonspecific GFP probes, we detected 6,837 *MEG3*-bound genomic regions associated with 5,622 genes (Table 2), as identified using the GREAT tool^37^. We found a significant overlap between the deregulated genes from the microarray experiment following *MEG3* downregulation and the genes associated with *MEG3* peaks (300 genes, *P*<5e^−14^, hypergeometric distribution), indicating a functional role of the *MEG3* peaks in the regulation of associated genes (Table 2, Supplementary Fig. 6b, Supplementary Data 8 and Supplementary Data 9). We also observed a significant overlap between the deregulated genes from RNA sequencing or from both microarray and RNA sequencing experiments, and the genes associated with *MEG3* peaks (Supplementary Fig. 6b,c). When we performed network analysis with the 300 deregulated genes associated with the *MEG3* peaks, we found that TGF-β was one of the major affected pathways (Supplementary Fig. 7a,b). The majority of the *MEG3*-bound peaks associated with the deregulated genes were located distal to the promoter, including genes involved in the TGF-β pathway (Table 2, Fig. 4b and Supplementary Fig. 8a-o), suggesting that the *MEG3*-bound regions may serve as distal regulatory elements, and that the *MEG3*/EZH2 functional interaction contributes to their regulation. To verify whether the enrichment of the *MEG3*-bound regions is due to an artefact of direct interaction between the *MEG3* genomic locus and the *MEG3* peaks identified, we performed ChOP using sense and antisense oligos. We validated the enrichment of the *MEG3* peaks associated with the TGF-β pathway genes in ChOP pull-down with the antisense oligos but not with the sense oligos. The enrichment with antisense oligos was lost when the chromatin was pretreated with RNase A (Supplementary Fig. 9a). This further suggests that the pull-down with antisense oligos is mediated by *MEG3* RNA rather than being the result of technical artefacts. To identify *MEG3*-bound peaks that overlap with putative enhancers in BT-549 cells, we performed H3K4me1 ChIP-seq and overlapped the H3K4me1 peaks with *MEG3* peaks, and found that 662 *MEG3* peaks overlapped with H3K4me1 peaks (H3K4me1/*MEG3* peaks) in BT-549 cells (Table 2). The H3K4me1/*MEG3* peaks' associated genes showed a significant overlap with the genes that were deregulated upon downregulation of *MEG3* and had at least one associated *MEG3* peak (Table 2 and Supplementary Fig. 6c).

We observed a decrease in the enrichment of both H3K27me3 and EZH2 over the distal *MEG3*-bound peaks of the TGF-β pathway genes upon downregulation of *MEG3* (Fig. 4c and Supplementary Fig. 9b), suggesting that *MEG3* is required for PRC2 recruitment and H3K27me3 maintenance at the distal regulatory elements. We tested the enhancer activity of the *TGFBR1*-associated H3K4me1/*MEG3* peaks using the luciferase system and found a significant increase in the enhancer activity of the peaks in the *MEG3* shRNA-transduced cells compared with the control shRNA cells (Supplementary Fig. 9c). We performed the chromosome conformation capture (3C) assay to measure the long-range interactions between the upstream H3K4me1/*MEG3* peaks and the *TGFBR1* promoter. In our 3C experiment, we detected interaction between the upstream H3K4me1/*MEG3* peaks and the *TGFBR1* promoter. Interestingly, these interactions were enhanced in the *MEG3* shRNA-transduced cells compared with the control shRNA cells (Fig. 4d), indicating that the *MEG3*/PRC2 functional interaction could regulate the activity of the distal regulatory elements.

### *MEG3* targets the TGF-β pathway genes via GA-rich sequences

**Figure 5:**
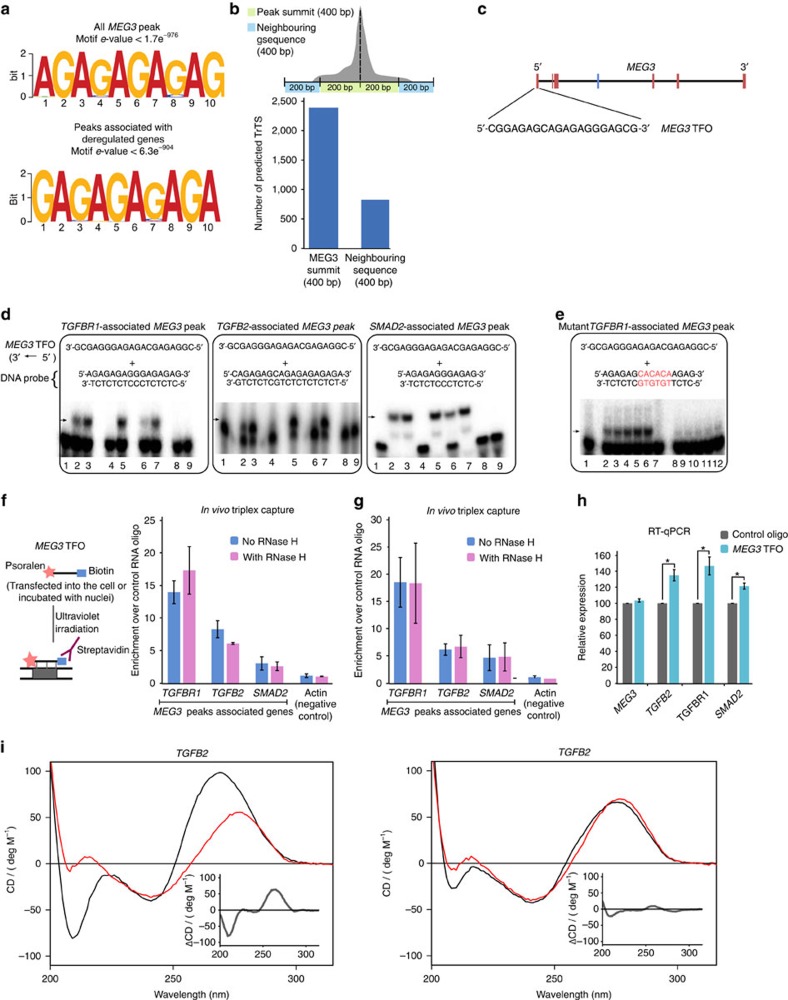
*MEG3* lncRNA regulates its target genes through triplex structure formation. (**a**) Predicted GA-rich motifs enriched in all *MEG3* peaks and peaks associated with deregulated genes using MEME-ChIP. (**b**) Number of TrTS over the *MEG3* peak summits and neighbouring regions, predicted by Triplexator^41^. (**c**) The schematic shows the *MEG3* TFO used in the triplex assays. The exons are colour-coded as described before. (**d**) Electrophoretic mobility shift assay. End-labelled dsDNA oligos (sequences provided in the schematic with gene name) were incubated alone (lane 1) or with increasing concentrations of *MEG3* ssRNA TFO (lanes 2 and 3: shift indicated with arrow) or with increasing concentrations of control ssRNA oligo (lanes 8 and 9). dsDNA oligos were incubated with *MEG3* TFO and treated with either RNase A (lane 4) or RNase H (lane 5). dsDNA oligos were incubated with *MEG3* ssRNA TFO in the presence of either unlabelled specific competitor (lane 6) or nonspecific competitor (lane 7). (**e**) *TGFBR1-*associated *MEG3* peak sequence and its mutated version (the changed nucleotides are in red) were incubated alone (lanes 1 and 7) or with *MEG3* TFO. Arrow indicates complex formation. (**f**,**g**) Enrichment of *MEG3* peak sequences using biotin- and psoralen-labelled *MEG3* TFO. RNase H-treated lysates were used to capture the labelled *MEG3* TFO using streptavidin beads. The enrichment of *MEG3* peaks is presented as the ratio between *MEG3* TFO and control oligo (±s.d., *n*=3). (**h**) RT–qPCR analysis of gene expression in BT-549 cells transfected with either *MEG3* TFO or control RNA oligo. Expression in *MEG3* TFO presented relative to the control oligo (±s.d., *n*=3). *P<0.05, Student's *t*-test (two-tailed, two-sample unequal variance). (**i**) Left panel: CD spectra of a 1:1 mixture of *TGFB2* dsDNA and *MEG3* TFO (ssRNA) are shown in black, and *TGFB2* dsDNA and the control ssRNA are shown in red. Right panel: the sum of the individual CD spectra for *TGFB2* dsDNA and *MEG3* TFO (ssRNA) is shown in black, and the sum of the individual CD spectra for *TGFB2* dsDNA and the control ssRNA is shown in red. Inset in the left and right panel shows the difference between the two spectra.

We next tried to investigate the mechanisms that facilitate how *MEG3* lncRNA selects its target regions across the genome. First, we looked for common sequence motifs enriched in the *MEG3*-bound genomic regions and identified a strong GA-rich sequence motif that was overrepresented among the 6,837 *MEG3* peak summits (motif *e*-value: 1.7e^−976^) (Fig. 5a). The GA-rich motif was also overrepresented among the 532 *MEG3* peaks (motif *e*-value: 6.3e^−904^) associated with the *MEG3*-deregulated genes (Fig. 5a), suggesting that the GA-rich repeat may play a functional role in targeting of the *MEG3* RNA to chromatin. Interestingly, by using the ChIRP technique, similar GA-enriched motifs were identified among the binding sites of the chromatin-modulating RNAs *roX2* and *HOTAIR*, indicating that GA-enriched motifs may play an important role in the targeting of lncRNAs across the genome^19^. Previously, several studies using different techniques have shown that GA-rich homopurine sequences can form triplex structures^38^,^39^,^40^. Overrepresentation of GA-rich sequences among the genomic binding sites of the lncRNAs analysed (*MEG3*, *HOTAIR* and *roX*) raises the possibility that the lncRNAs may be recruited to their target genes via RNA–DNA triplex formation. By using Triplexator software^41^ (which can predict triplex target sites, TrTS), we found a greater number of the predicted TrTS in the *MEG3* peak summit (±200 bp from the centre of the peak) than the flanking sequences (200 bp upstream and 200 bp downstream of the peak summit; Fig. 5b). Triplexator was also used to scan for triplex-forming oligonucleotides (TFOs) within the *MEG3* RNA, and several TFOs with high scores were detected. Interestingly, the TFOs with high scores are also enriched with GA-rich sequences (Table 3), indicating that the GA-rich sequences from target genes and *MEG3* RNA could form triplex structures by forming Hoogsteen bonds between RNA and DNA. To test the ability of *MEG3* lncRNA to form triplex structures, we used a 20-nucleotide-long GA-rich RNA oligo (hereon referred to as *MEG3* TFO) located at the 5′-end of the *MEG3* RNA and its sequence overlap with the TFOs (TFO1, TFO2 and TFO3) with high score that were identified by Triplexator (Fig. 5c and Table 3). Using electrophoretic mobility shift assay, we tested the triplex-forming ability of the *MEG3* TFO (single-stranded RNA, ssRNA) with the GA-rich (double-stranded DNA, dsDNA) *MEG3* peak sequences associated with the selected TGF-β pathway target genes (*TGFBR1*, *TGFB2* and *SMAD2*) *in vitro* (Fig. 5d). Consistent with the Triplexator predictions, we observed a shift in the end-labelled GA-rich dsDNA sequences when incubated with increasing concentrations of the *MEG3* TFO, indicating triplex formation between the *MEG3* TFO and the GA-rich *MEG3* peak summits (Fig. 5d, compare lane 1 with lanes 2 and 3), but not with a control RNA oligo selected from the *MEG3* lncRNA with no GA bias (Fig. 5d, compare lane 1 with lanes 8 and 9). The triplex structures were sensitive to RNase A treatment but were resistant to RNase H digestion (Fig. 5d, lanes 4 and 5, respectively), while an *in vitro* formed RNA–DNA hybrid was digested by RNase H (Supplementary Fig. 10a). These results together suggest that the observed shift was not because of Watson–Crick RNA–DNA pairing. We also observed that these shifts were affected when specific competitor (the same GA-rich dsDNA oligo, unlabelled) was used but were unaffected by nonspecific competitor (unlabelled control dsDNA oligo; Fig. 5d, compare lane 6 with 7). We did not observe any complex formation between *MEG3* TFO incubated with end-labelled control DNA sequences (with no GA bias) or control RNA incubated with control DNA sequences (Supplementary Fig. 10b,c). To further check the specificity of the interaction between *MEG3* TFO and GA-rich DNA sequences, we mutated the core sequences of the *TGFBR1*-associated *MEG3* peak and found that triplex formation was compromised between the mutant *TGFBR1* dsDNA oligo and the *MEG3* TFO (Fig. 5e). These observations suggest that *MEG3* may be recruited to genomic loci through the formation of RNA–DNA triplex structures. We predicted the triplex-forming ability of GA-rich motifs of another chromatin-interacting lncRNA, *HOTAIR*, and found more Triplexator-predicted TrTS from the *HOTAIR* summit regions than from the neighbouring sequences (Supplementary Fig. 10d,e).

We then investigated the formation of RNA–DNA triplex structures by using an alternative method whereby biotin-labelled *MEG3* TFO and a control RNA oligo were either used to transfect BT-549 cells (Fig. 5f) or incubated with nuclei isolated from BT-549 cells (Fig. 5g). Upon pull-down with streptavidin magnetic beads, we found significant enrichment of the selected *MEG3* peaks associated with the *TGF-β* genes—with *MEG3* TFO compared with control oligo. The enrichment of the *MEG3* target sequences was unaltered upon treatment with RNase H, suggesting that the interaction of the *MEG3* TFO with the target DNA sequence is not mediated by Watson–Crick RNA–DNA pairing (Fig. 5f,g). We next investigated whether the *MEG3* TFO occupancy at the *MEG3* target genes alters their transcriptional regulation. To this end, we analysed expression of the three key *TGF-β* genes *TGFB2*, *TGFBR1* and *SMAD2* in BT-549 cells after transfection with *MEG3* TFO or control RNA oligo. We found that expression of the TGF-β pathway genes was marginally, but significantly, upregulated in the *MEG3* TFO-transfected cells compared with the cells transfected with control oligo (Fig. 5h). These results indicate that the *MEG3* TFO sequence can compete with endogenous full-length *MEG3* RNA in binding to *MEG3* target sites, thus affecting the endogenous function of *MEG3* RNA.

We also performed circular dichroism (CD) spectroscopy to investigate the triplex-forming ability of some of the *MEG3* target sites with *MEG3* TFO. Figure 5i (left) shows the CD spectrum of the dsDNA oligo corresponding to the *TGFB*2-associated *MEG3* peak incubated with *MEG3* TFO ssRNA and the corresponding spectrum with a control ssRNA. The spectrum of the *MEG3* TFO sample has some distinct features, such as a distinct blue-shift (∼10 nm) of the peak ∼270–280 nm and a strong negative peak at ∼210 nm, which are not seen in the sample containing the control ssRNA. The effect is emphasized in the inset in Fig. 5i, where we show the difference in CD spectrum between the *TGFB*2 dsDNA oligo with the *MEG3* TFO and the control ssRNA. These two features, and especially the strong negative peak at ∼210 nm, are often seen for TFOs^42^,^43^,^44^. Figure 5i (right) shows the sum of the individual CD spectra for either *TGFB2* dsDNA and *MEG3* TFO (ssRNA) or *TGFB2* dsDNA and the control ssRNA. For these artificial spectra, the difference between the *MEG3* TFO and the control ssRNA is much smaller (Fig. 5i, right, inset), supporting our conclusion that the change in CD spectrum when *MEG3* TFO is incubated with the dsDNA *TGFB*2 is owing to a specific interaction between the two. We thus interpret the CD data, in combination with evidence from the complementary techniques, as being owing to the formation of a triplex structure between the dsDNA *TGFB*2 and the *MEG3* TFO ssRNA. CD spectra similar to the ones in Fig. 5i (left) were also detected with the *SMAD2*- (Supplementary Fig. 11a) and *TGFBR1* (Supplementary Fig. 11b)-associated *MEG3* peaks, indicative of triplex formation.

### Triplex structures are associated with *TGF-β* genes *in vivo*

**Figure 6:**
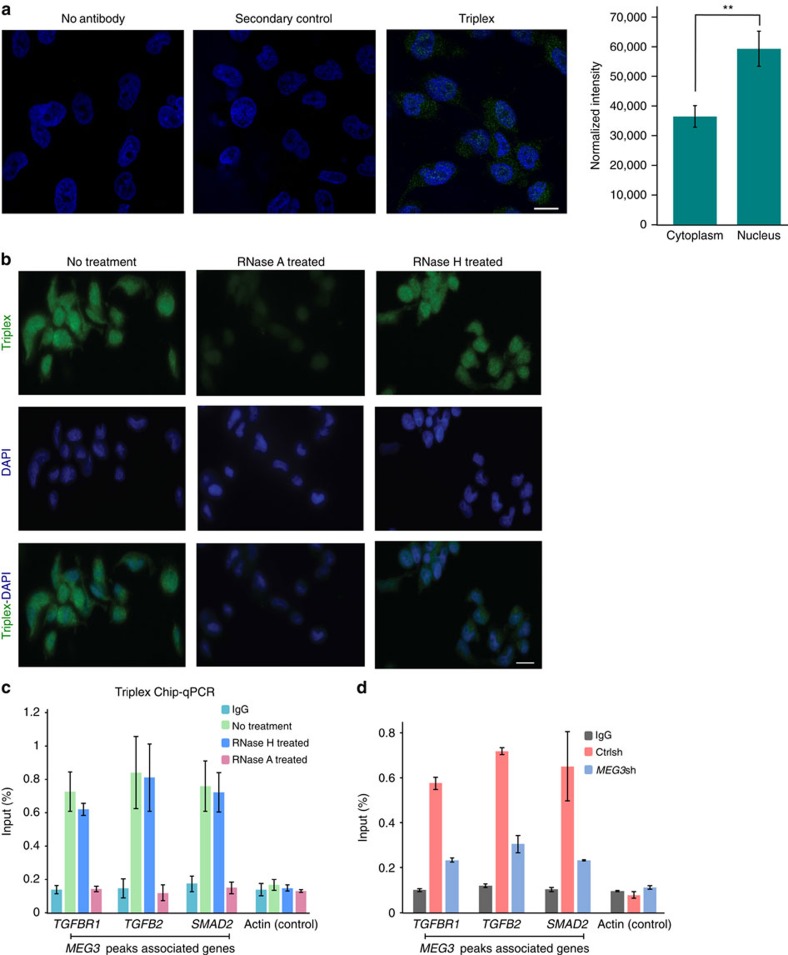
RNA–DNA triplexes are present *in vivo.* (**a**) Confocal microscopic images showing immunostaining with anti-triplex dA.2rU antibody (green) in BT-549 cells. The nucleus is stained with DAPI (4,6-diamidino-2-phenylindole; blue). Immunostaining with no antibody and secondary antibody were used as negative controls. Scale bar, 5 μm. The graph to the right shows quantification of the triplex signal in cytoplasm and nuclear compartments obtained from the three-dimensional confocal images. The graph represents the average of cytoplasmic and nuclear signals from >50 cells in several microscopic fields. The error bars indicate s.e.m. The *P* value was calculated using Student's *t*-test **P<0.01. (**b**) RNA–DNA triplex structures are sensitive to RNase A but are resistant to RNase H *in vivo*. Top panel: immunofluorescent staining of BT-549 cells with anti-triplex dA.2rU antibody (green) with no treatment (left), pretreated with RNase A (centre), or pretreated with RNase H (right) as indicated. Middle panel: cells were counterstained with DAPI (blue). Bottom panel: overlay of the triplex signals with DAPI staining. Scale bar, 5 μm. (**c**) Triplex-ChIP–qPCR showing enrichment (presented as percentage of input) of triplex structures over the *MEG3* peaks associated with the TGF-β pathway genes *(TGFBR1*, *TGFB2* and *SMAD2*) in BT-549 cells (±s.d., *n*=3). Actin was used as a negative control. Chromatin was pretreated with RNase A or RNase H before ChIP. Immunoglobulin G (IgG) was used as an antibody control. (**d**) Triplex-ChIP–qPCR showing enrichment (presented as percentage of input) of triplex structures over the *MEG3* peaks associated with the TGF-β pathway genes *(TGFBR1*, *TGFB2* and *SMAD2*) in Ctrlsh and *MEG3*sh BT-549 cells (±s.d., *n*=3). IgG was used as an antibody control.

Our *in vitro* triplex formation assay with *MEG3* RNA TFO and GA-rich *MEG3* target sequences suggests that RNA–DNA triplex formation could guide *MEG3* lncRNA to its target genes across the genome. This raises an intriguing question as to whether RNA–DNA triplex structures are present *in vivo*. In order to identify such triplex structures in BT-549 cells, we wanted to perform immunostaining with anti-triplex dA.2rU antibody, which can detect triplex structures. The specificity of the dA.2rU antibody in detecting triplex structures has been verified by enzyme-linked immunosorbent assay^45^,^46^. In addition, anti-triplex dA.2rU antibody has been used in immunostaining to detect the triplex structures on polytene chromosomes, and also in two-cell early pre-implantation mouse embryos^45^,^47^. Since the anti-triplex dA.2rU antibody was raised against the triplex derived from homopolymeric nucleic acids (poly(rU).poly(dA).poly(rU) complex)^46^, we wanted to test the ability of the anti-triplex dA.2rU antibody to recognize non-homopolymeric triplexes, which would be relevant for detecting triplex structures *in vivo*. For this, we performed immunodots with DNA triplexes built from poly-purine/poly-pyrimidine sequences, including controls for the antibody reactivity. As expected, the antibodies recognized homopolymeric triplexes containing a poly(dA) backbone and did not bind to nucleic acids when non-complementary sequences in solutions impeded formation of triple-stranded complexes. Also, the antibodies clearly bound to the triplex DNA made with poly-purine/poly-pyrimidine sequences (Supplementary Fig. 12a), indicating that antibody reactivity is not restricted to three-stranded configurations assembled with homopolymeric nucleic acids. To detect triplex structures *in vivo*, immunostaining was performed on BT-549 cells with anti- triplex dA.2rU antibody, and specific staining was observed with anti- triplex dA.2rU antibody (Fig. 6a). The anti-triplex staining was distributed in both the nucleus and the cytoplasm with more enrichment of the triplex-specific staining in the nuclear compartment (Fig. 6a). Triplex-specific staining was significantly reduced in the cells treated with RNase A (Fig. 6b, left and middle panel), but it was resistant to treatment with RNase H, which specifically cleaves RNA–DNA hybrids (Fig. 6b, right panel). The triplex staining pattern in BT-549 cells was similar to the staining of triplex structures that had been detected previously in human cells using a monoclonal anti-triplex antibody, Jel 318 (ref. 48 and Supplementary Fig. 12b). Although the triplex structures were more enriched in the nucleus, we detected triplex-specific staining in the cytoplasm, which could be due to recognition of the triplex structures present in mitochondria^49^,^50^. To test this, we labelled the mitochondria in BT-549 cells with MitoTracker followed by immunostaining with anti-triplex antibody. We indeed observed a co-localization of the mitochondrial staining with triplex signals from cytoplasm, suggesting that a part of the cytoplasmic triplex signals are contributed by the triplex structures present in mitochondria (Supplementary Fig. 12c). We next wanted to determine whether the triplex structures present at the *MEG3* binding sites are associated with the TGF-β pathway genes in BT-549 cells. We performed triplex-ChIP with anti-triplex dA.2rU antibody and observed enrichment of the selected *MEG3* peaks associated with the *TGFBR1*, *TGFB2* and *SMAD2* genes. To check the specificity of the anti-triplex dA.2rU pull-down, we pretreated the chromatin with either RNAse H or RNase A. RNase A treatment, but not RNase H, treatment resulted in complete loss of triplex enrichment (Fig. 6c). We also performed triplex-ChIP in *MEG3*-downregulated BT-549 cells and found a decrease in the enrichment of the triplex structures over the *MEG3* peaks associated with the *TGFBR1*, *TGFB2* and *SMAD2* genes, suggesting that the *MEG3* lncRNA regulate these genes through triplex formation (Fig. 6d).

### Chromatin-binding region of *MEG3* is functionally distinct

**Figure 7:**
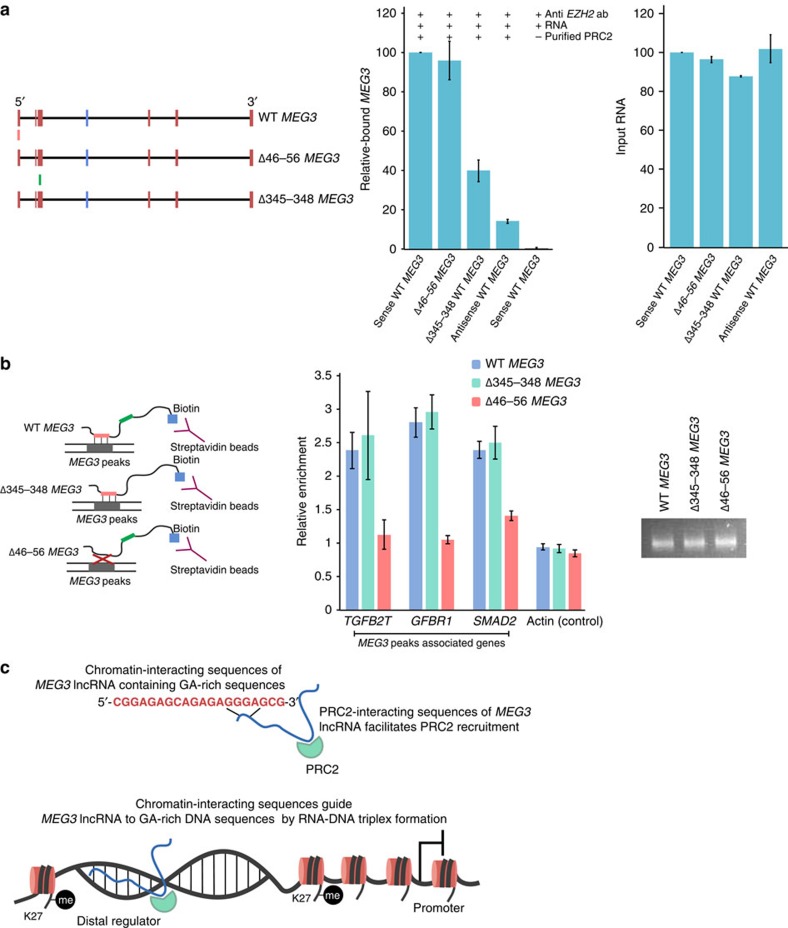
Chromatin-binding sequences and PRC2-binding sequences of *MEG3* lncRNA are functionally distinct. (**a**) *MEG3*-PRC2 *in vitro* binding assay. Left panel: schematic representation of WT *MEG3*, Δ46-56*MEG3* and Δ345-348*MEG3*. Green and red boxes indicate PRC2- and chromatin-interacting sequences, respectively. Middle panel: bar diagram showing the relative binding efficiency (as determined by RT–qPCR) of the sense WT *MEG3*, Δ46-56 *MEG3* and Δ345-348 *MEG3* RNAs in an *in vitro* PRC2-binding assay. Binding assays with no PRC2 and antisense WT *MEG3* served as negative controls. The PRC2-binding efficiency of sense WT *MEG3* was set to 100, and the binding efficiency of the *MEG3* mutants is presented relative to WT *MEG3* (±s.d., *n*=3). Right panel: RT–qPCR showing the quantification of the input sense WT *MEG3*, *MEG3* deletions (Δ340-348 or Δ345-348 MEG3) and antisense WT *MEG3* RNAs. (**b**) Deletion of *MEG3* TFO leads to loss of chromatin interaction. Left panel: schematic display of interaction of the WT *MEG3* and *MEG3* mutants (Δ46-56*MEG3* and Δ345-348*MEG3*) with the *MEG3* peak sequences *in vivo*. Red (*MEG3* TFO) and green (PRC2-interacting region) colour-coded regions indicate the location of the deleted *MEG3* RNA sequences 46-56 and 345-348, respectively. Biotin-labelled WT *MEG3* or *MEG3* mutants were used to transfect BT-549 cells followed by crosslinking with formaldehyde. RNAse H-treated cell lysates were incubated with streptavidin beads to capture the *MEG3* RNA-associated DNA. Middle panel: qPCR data are presented as the ratio of captured DNA in WT *MEG3* or *MEG3* mutants to captured non-biotinylated *MEG3* RNA (±s.d., *n*=3). Right panel: agarose gel picture showing the quality of the biotin-labelled WT and mutant *MEG3* RNAs (500 ng of each biotin-RNA was loaded). (**c**) Model depicting how chromatin-interacting sequences of *MEG3* lncRNA-containing GA-rich sequences form RNA–DNA triplex with the GA-rich DNA sequences to guide *MEG3* lncRNA to chromatin. PRC2-interacting sequences of *MEG3* lncRNA facilitate recruitment of the PRC2 to distal regulatory elements, thereby establishing H3K27me3 marks to modulate gene expression.

We wanted to determine whether the *MEG3* RNA sequences required for the PRC2 interaction and RNA–DNA triplex formation are functionally distinct. For this purpose, we generated a *MEG3* mutant by deleting the core *MEG3* TFO (the TFO used in the triplex assay above) containing GA-rich sequences and named this Δ46-56 *MEG3* (46–56 indicates the position of the nucleotides with respect to the 5′-end of *MEG3*). We found that the TFO deletion had no effect on the interaction of PRC2 with *MEG3* (Fig. 7a). Considering that Δ345-348 *MEG3* affects the PRC2 interaction of WT *MEG3* (Figs 7a and 2e), we decided to test the chromatin-interacting property of the WT and *MEG3* RNAs with deletions (Δ46-56 *MEG3* and Δ345-348 *MEG3*) by transfecting BT-549 cells with *in vitro*-synthesized biotin-labelled WT or mutant *MEG3* RNAs (Δ46-56 *MEG3* or Δ345-348 *MEG3*). We found that the WT and Δ345-348 *MEG3* RNAs could pull-down *MEG3* peak sequences associated with the *TGF-β* genes (*TGFB2*, *TGFBR1* and *SMAD2*), but not when the pull-down was performed with the Δ46-56 *MEG3* TFO deletion (Fig. 7b). This suggests that the decrease in the association between *MEG3* and PRC2 does not have any effect on the chromatin-binding property of *MEG3* RNA. Taken together, these observations indicate that the chromatin targeting and PRC2 interaction properties of *MEG3* lncRNA are mediated by distinct RNA sequences (Fig. 7c).

## Discussion

Previous studies have identified thousands of lncRNAs that interact with repressive chromatin modifiers such as EZH2 (refs 24, 25). The interaction of lncRNAs with chromatin modifiers suggests that lncRNAs may have a role in targeting the chromatin modifiers to chromatin. LncRNA-mediated recruitment of the chromatin modifiers to chromatin is exemplified by *Kcnq1ot1* and *HOTAIR* lncRNAs, which have been shown to interact with chromatin and recruit repressive chromatin modifier EZH2 (refs 10, 19, 23, 51). This raises the possibility that there could be many more lncRNAs that interact with chromatin and serve as a link between chromatin and chromatin modifiers. Use of antibodies to EZH2 and its catalysed repressive chromatin mark H3K27me3 in our ChRIP-seq enabled us to identify 276 chromatin-interacting lncRNAs that are enriched in both EZH2 and H3K27me3 purified chromatin fractions. We expect that these chromatin-interacting lncRNAs will be a valuable resource for future investigations aimed at understanding the molecular mechanisms that dictate association of lncRNAs with chromatin. Indeed, by using one of the repressive chromatin-interacting lncRNAs, *MEG3*, we have characterized the mechanisms by which *MEG3* lncRNA is guided to chromatin. Deciphering of the mechanisms that guide the EZH2-interacting lncRNAs to repressive chromatin will also shed light on how the PRC2 complex is targeted across the genome in an RNA-dependent manner. Repressive chromatin-associated lncRNAs also include several lncRNAs (*PCA3*, *GAS6-AS1*, *CECR7* and *BDNF-AS1*) that have been implicated in cancer or other cellular functions, and among this *BDNF-AS1* lncRNA has been shown to regulate gene expression by recruiting PRC2 (refs 30, 52, 53, 54).

Mapping of the *MEG3* binding sites across the genome in BT-549 cells revealed that the majority of *MEG3* target sites are located distal to the promoter regions. Interestingly, a significant proportion of the promoter–distal *MEG3* binding sites are enriched with enhancer chromatin marks. More importantly, we observed that the interaction between the *TGFBR1* gene promoter and a putative enhancer increased significantly in the absence of *MEG3*. In addition, the downregulation of *MEG3* also resulted in loss of EZH2 and H3K27me3 enrichment at the putative enhancer. These results suggest that the *MEG3* lncRNA modulates the activity of the putative enhancer by regulating chromatin structure, thereby fine-tuning gene expression.

We detected over 6,800 *MEG3* binding sites, and a proportion of these *MEG3* peaks were associated with the genes that were either upregulated or downregulated after *MEG3* knockdown in BT-549 cells. Given the association of *MEG3* with a repressive chromatin modifier, EZH2, one would expect that the upregulated genes are more direct targets of the *MEG3*/EZH2 interaction. On the other hand, *MEG3* peaks also flanked the genes that showed repression in the absence of *MEG3*. We suggest that the expression of these genes may be facilitated by the recruitment of EZH2 by *MEG3*, and this EZH2-dependent activation of genes was evident in recent findings where EZH2 has been shown to act as a coactivator of gene expression in prostate and breast cancer cells^55^,^56^. Although *MEG3*-regulated genes were enriched in the *MEG3* peaks, there was no one-to-one correlation between the *MEG3* peaks and associated genes. This could be explained in part by the non-reversible nature of noncoding RNA-mediated chromatin modification; that is, once a chromatin mark is established in an RNA-dependent manner, it is maintained in the absence of the RNA^57^. The stable maintenance of RNA-mediated repressive modification after establishment was also observed with *Kcnq1ot1* lncRNA-mediated silencing, where the RNA was removed conditionally after the repressive chromatin was established^58^. It is possible that a similar mechanism exists in *trans*-acting lncRNAs such as *MEG3*, where RNAi-mediated knockdown of the lncRNA does not lead to deregulation of all its target genes because of RNA-independent maintenance of the chromatin structure.

We have explored the molecular mechanisms by which *MEG3* lncRNA contributes to regulation of the TGF-β pathway. We found that the genes of the TGF-β pathway are direct targets of *MEG3*, and that it regulates these genes by binding to promoter–distal regulatory regions. Consistent with our results, a recent investigation has found that *MEG3* expression was downregulated upon TGF-β1 treatment in human hepatic stellate cells, and that *MEG3* overexpression inhibited the TGF-β1-stimulated cell proliferation and induced apoptosis^59^. These observations together with our data indicate the existence of a probable feedback loop between the *MEG3* and TGF-β pathway. This target recognition by *MEG3* occurs via triplex formation between GA-rich sequences of target genes and GA-rich sequences within *MEG3* lncRNA. Interestingly such as *MEG3*, the *HOTAIR* binding sites also have GA-rich sequences^19^. Formation of triplex structures between *MEG3* lncRNA and GA-rich sequences in our triplex assays indicates that GA-rich sequences may guide lncRNAs to their target genes. Immunostaining with monoclonal antibody to triplex structures revealed that these structures are widespread *in vivo*. Furthermore, using Triplex-ChIP assay, we found that triplex structures were present *in vivo* over the *MEG3* peaks associated with the TGF-β pathway genes. Taken together, these observations further suggest that targeting of *MEG3* lncRNA to chromatin occurs through RNA–DNA triplex formation. Our observation on the mode of the chromatin targeting of *MEG3* through RNA–DNA triplex formation along with the previous evidence of triplex-mediated communication of lncRNAs with their target genes suggests that this type of mechanism may be more general^38^,^60^,^61^,^62^,^63^. Similar to the *trans*-acting role of human *MEG3* in breast cancer cells, a recent investigation by Kaneko *et al.*^29^ demonstrated that interaction between JARID2 and *MEG3* lncRNA is critical for targeting of PRC2 complexes to multiple genes *in trans* in mouse embryonic stem cells. As JARID2 has also been found to play a critical role in activating the catalytic function of PRC2 by weakening the RNA–PRC2 interaction, it would be interesting to investigate whether JARID2 has any such role in regulation of human *MEG3*–PRC2 interaction as well^64^. In this context, our study is particularly significant, as it contributes to our understanding of the mechanisms underlying the lncRNA-mediated targeting of PRC2 complex across the genome.

Functional overlap between *MEG3-* and *EZH2-*deregulated genes, and mapping of a significant number of *MEG3* binding sites to *MEG3*-deregulated genes indicate that *MEG3* has a functional role in guiding PRC2 to its target genes across the genome. Fine-mapping of *MEG3* RNA sequences required for PRC2 interaction and chromatin targeting via triplex formation suggests that while the triplex-forming sequences may guide *MEG3* lncRNA to chromatin, the PRC2-interacting sequences facilitate the recruitment of PRC2 to promoter–distal regulatory regions, thereby depositing H3K27me3 to modulate transcriptional activity (Fig. 7c). Our data on *MEG3* RNA together with the published data on *HOTAIR* indicate that the GA-rich homopurine motif may be the preferred binding site for both *MEG3* and *HOTAIR* lncRNAs. Interestingly, the GA-rich motif is also present in *Drosophila*^65^,^66^, *Arabidopsis*^67^ and mammalian^68^,^69^ polycomb response elements. In *Drosophila melanogaster,* PRC2 recruitment to the GA-rich motif has previously been shown to occur via a DNA-binding transcription factor, but no such factor has been characterized in mammals^65^. GA-rich motifs may be preferred sequences for RNA-dependent PRC2 recruitment, and thus lncRNAs may bypass the requirement for protein factors in PRC2 recruitment.

## Methods

### Molecular cloning

Full-length *MEG3* cDNA was amplified from BT-549 nuclear RNA and cloned into either pCMV6-XL5 (OriGene) or pREP4 episomal vector (Life Technologies) using the primers described in Supplementary Data 10. The details of the exon compositions of the full-length *MEG3* clone are provided in the Results section. Mutant *MEG3* RNAs (Δ340-348, Δ345-348 and Δ46-56 *MEG3*) described in the manuscript were generated using the Quik-change site-directed mutagenesis kit (Agilent Technologies). Primers used in the site-directed mutagenesis and for cloning of *MEG3* are provided in Supplementary Data 10. For *in vitro* transcription of biotin-labelled and unlabelled *MEG3* RNA, full-length *MEG3* (referred to as WT *MEG3*) or the mutant *MEG3* RNAs, carrying various deletions, were cloned into pGEM-T Easy vector (Promega).

### Transfection and RT–qPCR assay

siRNAs were used for transfection using Lipofectamine RNAiMAX reagent (Life Technologies). For each siRNA transfection, 50,000 BT-549 or HF cells were seeded per well in 24-well plates 12–16 h before transfection. siRNAs against *MEG3* and *EZH2* or the control siRNA were used for transfection at a final concentration of 65 nM (details of siRNAs are provided in Supplementary Data 10). Forty-eight hours after transfection, RNA was isolated using the ReliaPrep RNA isolation kit (Promega). DNase I-treated RNA (500 ng) was converted to cDNA using reverse transcriptase (Promega) and assayed for gene expression by SYBR Green-based RT–qPCR using the Vii7 Real-Time PCR system (Life Technologies) with the relevant primers listed in Supplementary Data 10. A negative control reaction without any cDNA was included in every qPCR. Episomal pREP4 plasmids (only pREP4 vector or pREP4 containing *MEG3*) were used to transfect BT-549 cells using Lipofectamine 2000 reagent (Life Technologies) and overexpression of *MEG3* RNA was verified by RT–qPCR 48 h after transfection, using primer pairs overlapping *MEG3* exon 3 (primer sequences are provided in Supplementary Data 10). pCAG-h*EZH2* or the control DNA plasmid was used to transfect BT-549 cells using Lipofectamine 2000. *EZH2* overexpression was confirmed by RT–qPCR 48 h after transfection, using primer pairs provided in Supplementary Data 10.

### Cell culture and generation of stable clones

BT-549 cells were maintained in RPMI (Sigma) supplemented with 10% fetal bovine serum (FBS) (Sigma). MDA-MB-231 and HF cells were maintained in DMEM (Invitrogen) with 10% FBS. BT-549 cells were procured from CLS cell line service. MDA-MB-231 cells were kindly gifted by Dr Briegel Karoline (University of Miami Miller School of Medicine, Miami, USA) and HF cells were kindly gifted by Dr Bengt Westermark (Uppsala University, Uppsala, Sweden). MISSION lentiviral transduction particles expressing non-target shRNA control or shRNA against *MEG3* were obtained from Sigma (the sequences are provided in Supplementary Data 10) and were used for transduction following the manufacturer's protocol. BT-549 cells (5 × 10^4^) were plated in the wells of 24-well plates and transduced with either control or *MEG3* shRNA lentiviral particles. Lentivirus-transduced BT-549 cells were selected with puromycin to obtain stably integrated shRNA vectors. The stable clones were maintained thereafter in RPMI containing puromycin (1 μg ml^−1^). We verified the downregulation of the *MEG3* lncRNA in lentivirus-transduced stable clones by RT-qPCR. Stable clones of MDA-MB-231 cells containing pREP4*MEG3* were generated after antibiotic selection with hygromycin (850 μg ml^−1^), and maintained in DMEM containing hygromycin (850 μg ml^−1^). We verified the overexpression of the *MEG3* RNA in MDA-MB-231 stable clones by RT–qPCR following the same procedure as described above.

### ChRIP-seq

ChRIP was performed using BT-549 cells adapting the protocols from Mondal *et al.*^28^ and Kuo *et al.*^70^ with the modifications as detailed below. BT-549 cells were plated on tissue culture dish (7–8 million cells per 150 mm plate) and incubated overnight (14–16 h) with 4sU (Sigma) at a final concentration of 100 μM in cell culture media. Next day, ActD (5 μg ml) was added to the media and incubated for 40–45 mins. To check the efficacy of ActD treatment, cells were incubated with and without ActD, and RNA was extracted and assayed level of *c-Myc* RNA, with a short half-life, by RT–qPCR. After ActD incubation, cells were washed two times with PBS and crosslinked with 1% formaldehyde for 10 min with gentle shaking. Crosslinking was stopped by adding glycine to a final concentration of 125 mM and incubated for 5 min with gentle shacking. Cells were subsequently washed twice with PBS followed by crosslinking on ice with ultraviolet. Cells were removed by scraping from the plate and resuspended in cold PBS. Nuclei were isolated using 1 × nuclei isolation buffer (40 mM Tris-HCl (pH 7.5), 20 mM MgCl_2_, 4% Triton X-100 and 1.28 M sucrose) and washed again with PBS. The isolated crosslinked nuclei were resuspended in lysis buffer (0.1% SDS, 0.5% Triton X-100, 20 mM Tris-HCl (pH 7.5), 150 mM NaCl and 1 ml lysis buffer per 10 million of cells) supplemented with RNasein (Promega) and subjected to sonication (Bioruptor, 20-30 cycles) to obtain chromatin fragments of ∼1 kb. An amount of 50–60 μg of soluble chromatin was used in each chromatin immunoprecipitation and incubated with 5 μg of anti-EZH2 (active motif) and anti-H3K27me3 (Merck-Millipore) antibody. Antibody-bound chromatin was washed according to our earlier published protocol with buffers and was supplemented with RNasein. Protein A magnetic beads bound to immunoprecipitated chromatin were resuspended in 10 × volume of elution buffer (100 mM NaCl, 10 mM Tris (pH 7.5), 1 mM EDTA and 0.5% SDS) containing proteinase K. Proteinase K treatment was carried out at 55 °C for 45 min followed by heating at 95 °C for 10 min to reverse crosslinking. Chromatin-bound RNA was extracted with Trizol (Life Technologies) and subjected to DNase I (Promega) treatment to remove traces of DNA. Since the chromatin-bound RNA yield from one single experiment is suboptimal for high-throughput sequencing, we pooled RNA from 6 to 8 ChRIP pull-downs, and the sequencing library was made using SOLiD Total RNA-Seq Kit and sequenced the library using SOLiD platform (Applied Biosystem). SOLiD Total RNA-Seq Kit allows obtaining strand-specific RNA sequencing information. Since library preparation in SOLID protocol involves ligation of RNA adaptors to the RNA fragments by RNA ligase, no contamination from DNA fragments is expected. For input, nuclear RNA was isolated from 4sU- and ActD-treated BT-549 cells, and depleted ribosomal RNA using RiboMinus Eukaryote System v2 (Life Technologies). For ChRIP validation, we followed the same protocol as described above with or without ActD treatment using antibodies against H3K27me3, EZH2, H3K4me2 (Merck-Millipore) and nonspecific Rabbit IgG (immunoglobulin G; Merck-Millipore).

## Additional information

**Accession codes:** The data associated with this publication have been deposited in European Nucleotide Archive and are accessible through accession number PRJEB7307.

**How to cite this article:** Mondal, T. *et al.* *MEG3* long noncoding RNA regulates TGF-β pathway genes through formation of RNA–DNA triplex structures. *Nat. Commun.* 6:7743 doi: 10.1038/ncomms8743 (2015).

## Supplementary Material

Supplementary Information Supplementary Figures 1-13, Supplementary Methods and Supplementary References

Supplementary Data 1 List of the annotated and non-annotated lncRNAs enriched in H3K27me3

Supplementary Data 2 List of the annotated and non-annotated lncRNAs enriched in EZH2

Supplementary Data 3 List of the annotated and non-annotated lncRNAs enriched in both H3K27me3 and EZH2

Supplementary Data 4 List of repressive chromatin-enriched annotated and non-annotated lncRNAs with T to C conversion

Supplementary Data 5 The deregulated genes as detected by microarray after MEG3 and EZH2 downregulation in BT-549 cells and the overlap between the MEG3 and EZH2-dependent genes

Supplementary Data 6 The deregulated genes as detected by microarray after MEG3 and EZH2 downregulation in HF cells and the overlap between the MEG3 and EZH2-dependent genes

Supplementary Data 7 List of pathways commonly affected after MEG3 and EZH2 downregulation in BT-549 cells, as detected by both microarray and RNA-seq

Supplementary Data 8 Summary of MEG3 peaks associated with the deregulated genes after MEG3 downregulation in BT-549 cells

Supplementary Data 9 Location and length of the MEG3 peaks associated with the deregulated genes after MEG3 downregulation in BT-549 cells

Supplementary Data 10 List of the oligos used in the study

## Figures and Tables

**Table 1:** KEGG pathway analysis of the deregulated genes identified by microarray and RNA-sequencing after downregulation of *MEG3* and *EZH2* by siRNA in BT-549 cells using GeneSCF.

**KEGG-ID**	**KEGG pathways**	***MEG3*** **(** ***P*** **value)**		***EZH2*** **(** ***P*** **value)**	
		**Microarray**	**RNA-seq**	**Microarray**	**RNA-seq**
hsa05200	PI3K-Akt signalling pathway	0.0000000307	0.00036	0.0001	0.00000376
hsa04390	Proteoglycans in cancer	0.000000472	0.00494	0.00000787	0.0021
hsa05166	Pathways in cancer	0.00000099	0.000000697	0.00000808	0.002
hsa04350	TGF-beta signalling pathway	0.0000059	0.0000893	0.00000062	0.0002
hsa05205	HTLV-I infection	0.00000865	0.00000239	0.0002	0.0432
hsa04151	Focal adhesion	0.0000188	0.00076	0.0005	0.0000239
hsa04910	Insulin signalling pathway	0.0000246	0.0175	0.0087	0.0274
hsa04510	Hippo signalling pathway	0.0000867	0.000002029	0.0001	0.0022
hsa01100	Colorectal cancer	0.0001	0.0017	0.000029	0.0009
hsa05414	Regulation of actin cytoskeleton	0.0003	0.0001	0.0012	0.0000114
hsa04010	Endocytosis	0.0005	0.0000187	0.0002	0.0002
hsa00532	Pancreatic cancer	0.0008	0.01	0.0002	0.0000439
hsa04668	Hypertrophic cardiomyopathy	0.0013	0.00000569	0.0053	0.0238
hsa05203	Chagas disease	0.0013	0.0028	0.000022	0.0054
hsa04520	Adherens junction	0.0016	0.0002	0.000079	0.0005
hsa05142	Viral carcinogenesis	0.0017	0.0144	0.0082	0.0177
hsa05410	TNF signalling pathway	0.0019	0.00107	0.0052	0.0001
hsa05212	Glycosaminoglycan biosynthesis	0.0027	0.002	0.0311	0.0136
hsa04144	MAPK signalling pathway	0.0062	0.0004	0.014	0.0001
hsa04810	Dilated cardiomyopathy	0.02	0.0000489	0.0013	0.0289
hsa05210	Metabolic pathways	0.04	0.0269	0.0009	0.0216

siRNA, small interfering RNA; TGF, transforming growth factor.

*P value represents Fisher's exact test and obtained using GeneSCF (see Methods section).

**Table 2:** Summary of *MEG3* peaks with associated genes and their overlap with H3K4me1 peaks in BT-549 cells.

**Summary of the** ***MEG3*** **binding sites in BT-549 cells**	**No. of peaks**	**No. of genes associated**
*MEG3* binding sites in *BT-549 cells*	6,837	5,622
Number of *MEG3* peaks in promoter	217	173
Number of *MEG3* peaks in promoter–distal region	6,620	5,449
Peaks associated with *MEG3*-deregulated genes (*MEG3*-deregulated peaks)	532	300
Number of *MEG3*-deregulated peaks in distal	524	292
Distal *MEG3* peaks overlap with BT-549 H3K4me1 peaks	662	959
Distal *MEG3*-derugulated peaks overlap with BT-549 H3K4me1 signals	56	52

**Table 3:** TFOs predicted by Triplexator.

**OligoID**	**TFOs (5′–3′)**	**Score**
TFO1	**GGAGAGcAGAGAGGGAGcG**	18
TFO2	GG**cGGAGAGcAGAGAGGGAGcG**	19
TFO3	AGAcGG**cGGAGAGcAGAGAGGGAG**	21
TFO4	AGGAtGGcAAAGGAtGAAGAGGA	20
TFO5	AAAtGAGAtAAAAGAGG	15
TFO6	GTcTTTGcTTGTGTT	13
TFO7	TGGGTGGGcTTcTGG	13
TFO8	TaGGGTTGTTGTGaG	13
TFO9	GGGcTGTTGTGaGGGG	14

TFOs, triplex-forming oligos; Score, triplex-forming potential scores.

The bold nucleotides indicate overlap with *MEG3* TFO with high score.
