# Author Correction: *MEG3* long noncoding RNA regulates the TGF-β pathway genes through formation of RNA–DNA triplex structures

**DOI:** 10.1038/s41467-019-13200-7

**Published:** 2019-11-21

**Authors:** Tanmoy Mondal, Santhilal Subhash, Roshan Vaid, Stefan Enroth, Sireesha Uday, Björn Reinius, Sanhita Mitra, Arif Mohammed, Alva Rani James, Emily Hoberg, Aristidis Moustakas, Ulf Gyllensten, Steven J. M. Jones, Claes M. Gustafsson, Andrew H. Sims, Fredrik Westerlund, Eduardo Gorab, Chandrasekhar Kanduri

**Affiliations:** 1 Department of Medical Genetics, Institute of Biomedicine, The Sahlgrenska Academy, University of Gothenburg, SE-40530 Gothenburg, Sweden; 2 Department of Immunology, Genetics and Pathology, Biomedical Center, SciLifeLab Uppsala, Uppsala University, SE-75108 Uppsala, Sweden; 3 Department of Medical Biochemistry and Cell Biology, University of Gothenburg, PO Box 440, SE-405 30, Gothenburg, Sweden; 4 Department of Medical Biochemistry and Microbiology, Science for Life Laboratory, Uppsala University, PO Box 582, SE-751 23 Uppsala, Sweden; 5 Ludwig Institute for Cancer Research, Science for Life Laboratory, Uppsala University, PO Box 595, SE-751 24 Uppsala, Sweden; 6 Genome Sciences Centre, British Columbia Cancer Agency, Vancouver, British Columbia BC V5Z 4S6 Canada; 7 Applied Bioinformatics of Cancer, University of Edinburgh Cancer Research UK Centre, Edinburgh, EH4 2XR UK; 8 Department of Chemical and Biological Engineering, Chalmers University of Technology, Gothenburg, 412 96 Sweden; 9 Departamento de Genética e Biologia Evolutiva, Instituto de Biociências, Universidade de São Paulo, São Paulo, CEP:05508-090 Brazil

Correction to: *Nature Communications* 10.1038/ncomms8743, published online 24 July 2015.

This article contains an error in Fig. 3g. The image in the Ctrlsh+TGF-β ligand panel was inadvertently duplicated from the image in the MEG3sh panel. The correct version of this figure appears below as Fig. 1.

**Figure 1:**
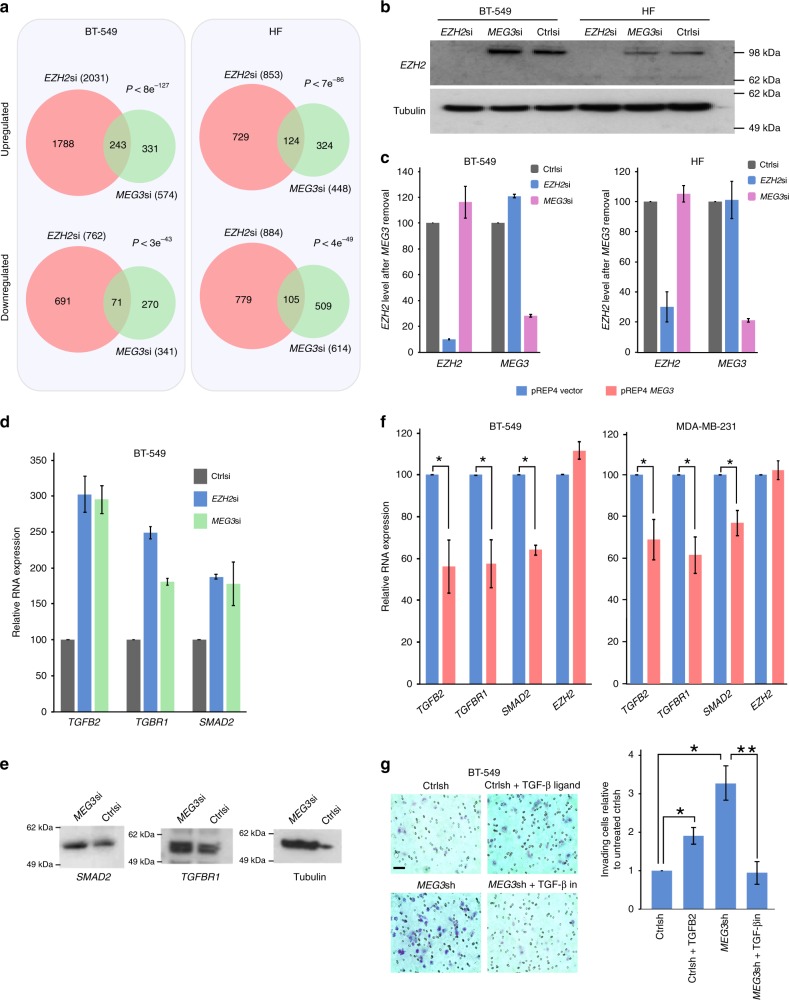
Fig. 1

