# Supplementary material for: MEG3 long noncoding RNA regulates the TGF-β pathway genes through formation of RNA–DNA triplex structures
Source: Nat Commun. 2015 Jul 24;6:7743. doi: 10.1038/ncomms8743 (PMC4525211; doi:10.1038/ncomms8743)
Supplement: Supplementary Information — Supplementary Figures 1-13, Supplementary Methods and Supplementary References [file ncomms8743-s1.pdf]

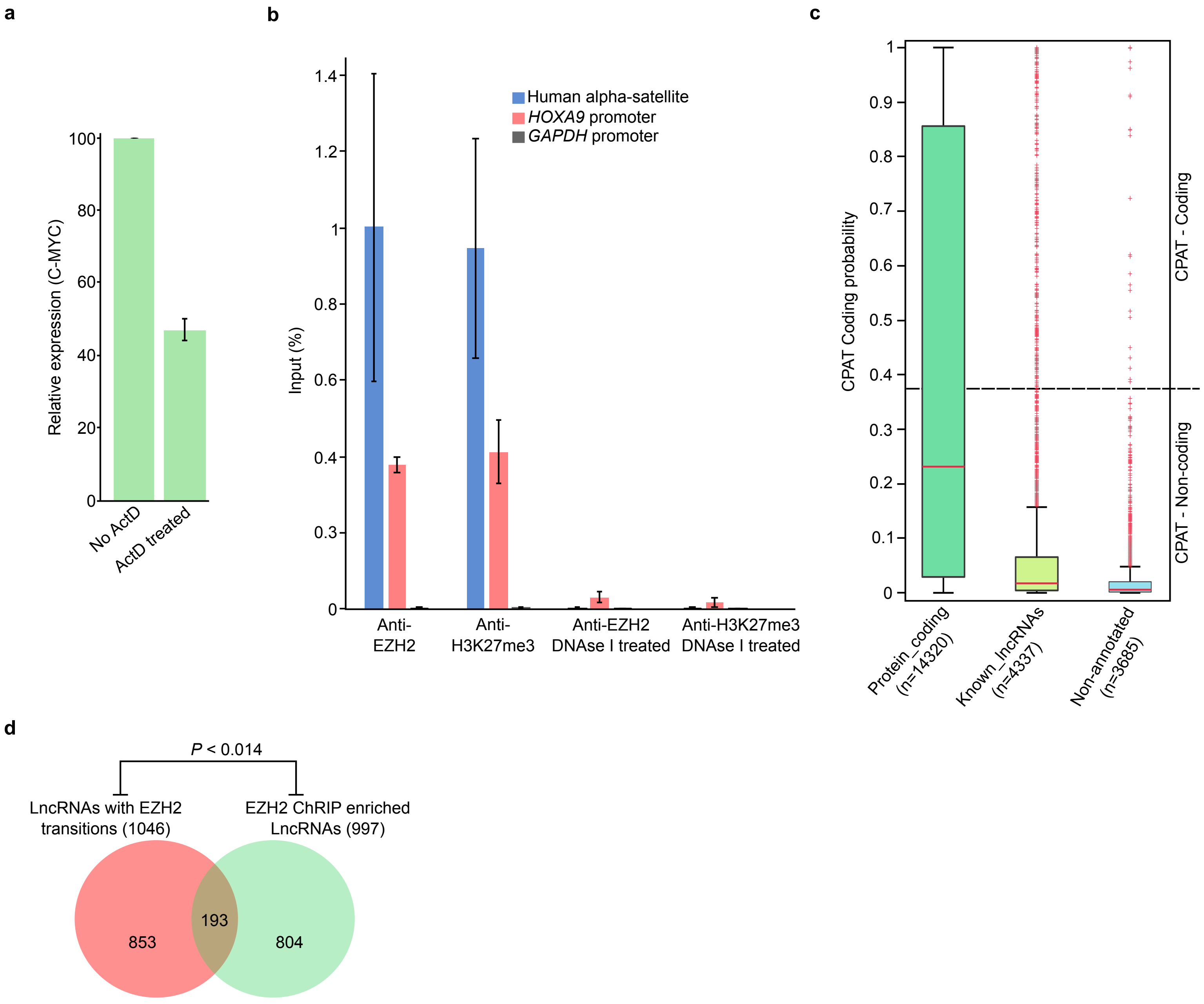

Supplementary Figure 1. Data related to ChRIP.

a. RT-qPCR analysis showing expression of the *C-MYC* in actinomycin D- (ActD-) treated or untreated BT-549 cells. *C-MYC* expression in treated and untreated cells was normalized to *GAPDH* expression.

b. ChIP-qPCR result showing enrichment of EZH2 and H3K27me3 chromatin marks, represented as percentage of input, over the human alpha satellite repeats and the *HOXA9* promoter, used as positive controls, in BT549 cells [1,2] . The GAPDH promoter was used as a negative control, and showed no enrichment in EZH2 and H3K27me3 ChIP. DNase I treatment resulted in loss of enrichment over the human alpha satellite repeats and *HOXA9* promoter.

c. Coding potential probability of non-annotated transcripts from the analysis using CPAT. Protein coding mRNAs and known lncRNAs were presented along with the non-annotated transcripts for the comparison. Most of the non-annotated transcripts had CPAT score less than 0.37 (CPAT score <=0.37 indicate non-coding).

d. 1,046 lncRNAs (annotated and non-annotated) were associated with EZH2-specific (17,625) T-to-C conversion. 193 EZH2 enriched lncRNAs (out of 997) carry T-to-C transitions. The *P* value was obtained by performing a hypergeometric test using all the lncRNAs considered in our analysis.

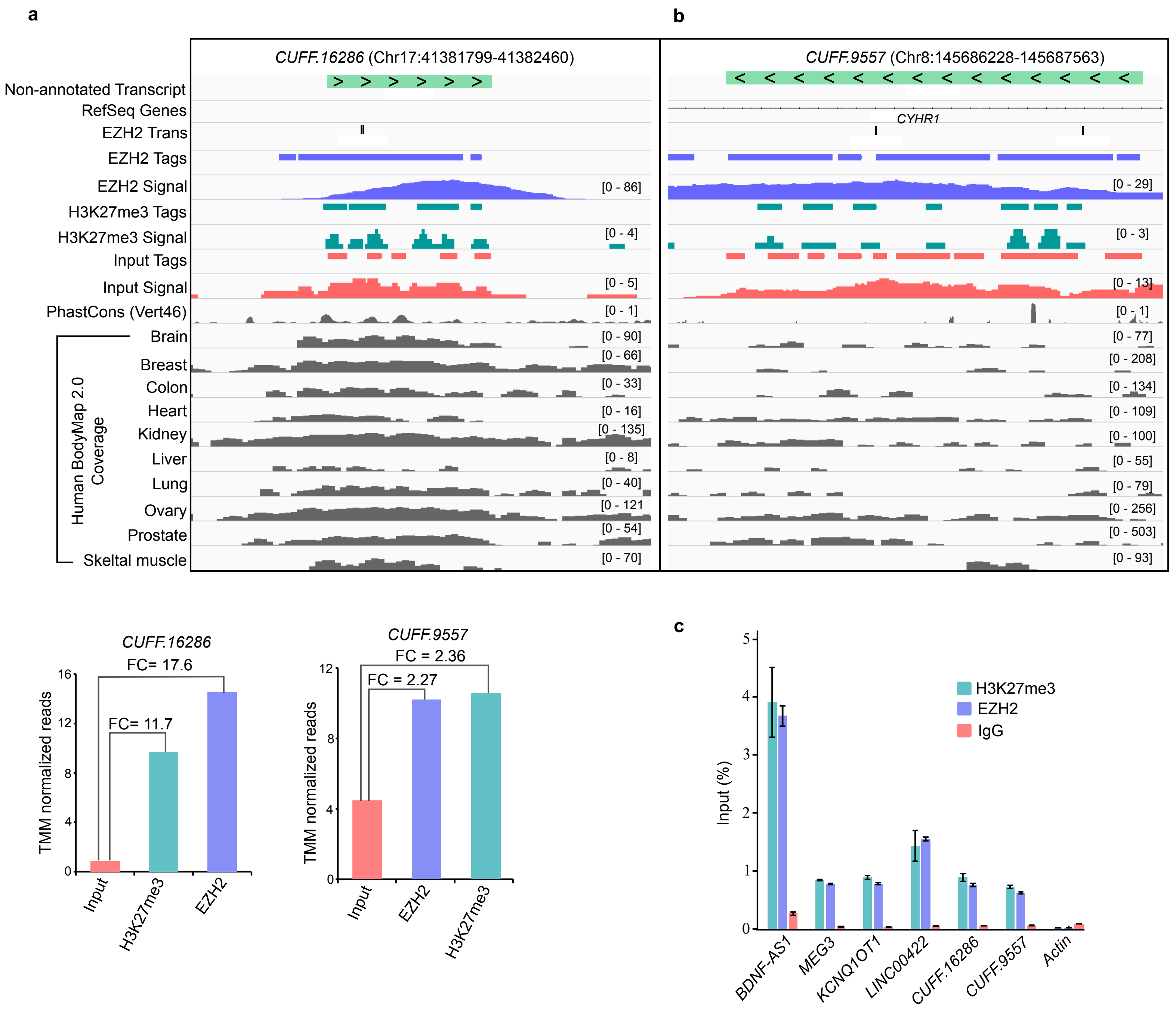

Supplementary Figure 2. ChRIP enriched non-annoated transcript and ChRIP validation.

a, b. Integrative Genomics Viewer showing the distribution of sequencing reads over a non-annotated intergenic transcript, *CUFF.16286*, and an intronic transcript, *CUFF.9557*, in H3K27me3, EZH2-enriched chromatin fractions and input RNA, respectively (data represent one biological replicate for each sample). The tags represent the read distribution and the signal represents the intensity (as indicated by the scale) over the transcripts. Locations of T-to-C transitions are depicted below the physical maps. The tracks also show phastCons scores, conservation across 46 vertebrate species, and RNA-seq coverage for 10 different tissues from Illumina's Human BodyMap 2.0 project. The panels below depict the RPKM values for *CUFF.16286* and *CUFF.9557* in H3K27me3, EZH2 ChRIP RNA, and input RNA samples. The fold enrichment (FC) in H3K27me3 and EZH2 ChRIP RNA compared to input is indicated.

c. ChRIP validation in actinomycin D untreated BT-549 cells: RT-qPCR data showing the enrichment of the selected annotated and non-annotated lncRNAs in the EZH2 and H3K27me3 ChRIP pulldowns compared to input. IgG was used as non-specific antibody in ChRIP pulldowns. *Actin* was used as a negative control. Data represent the mean ± SD of two independent biological experiments.

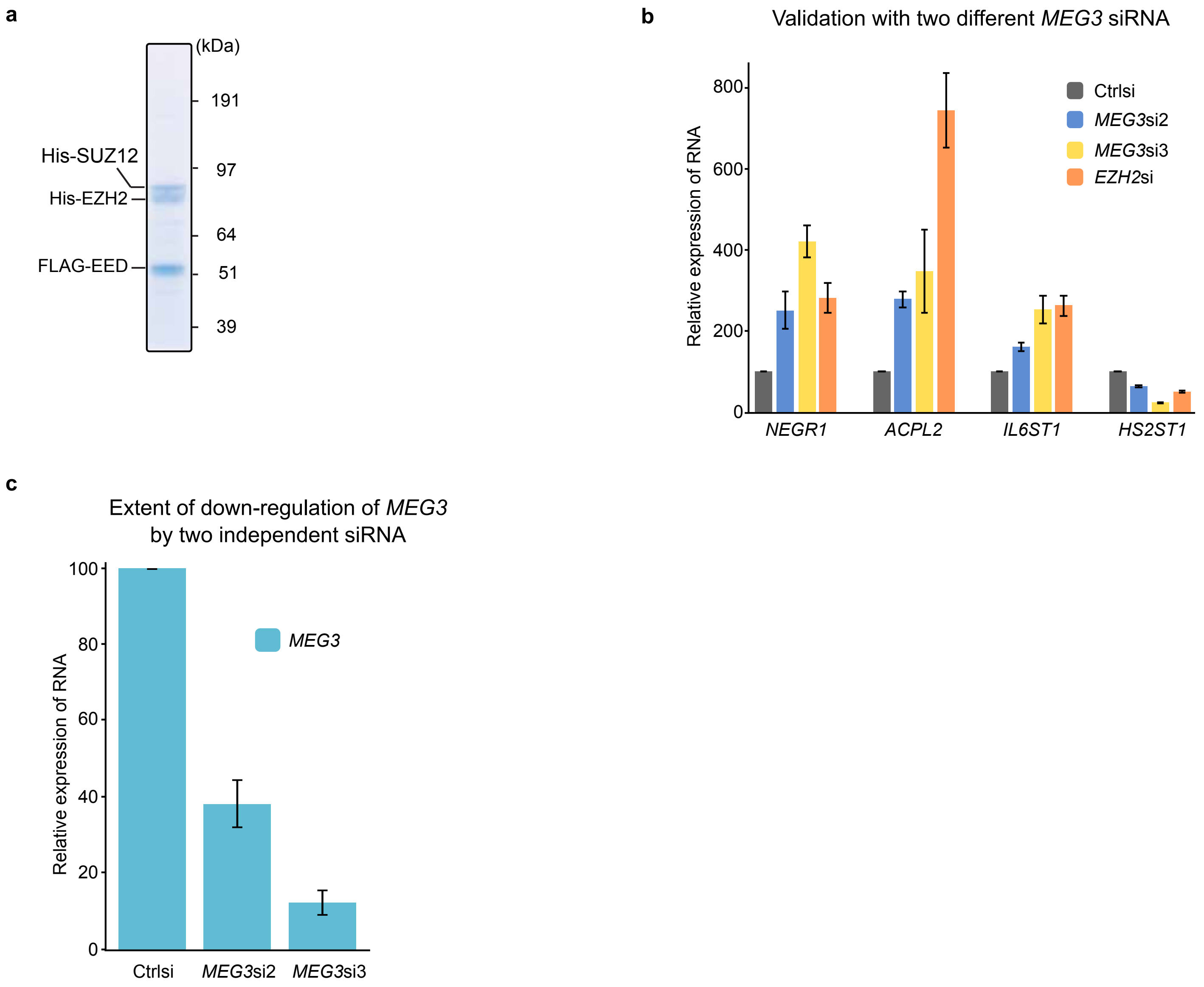

Supplementary Figure 3. Coomassie gel showing PRC2 and siRNA validation.

a. Coomassie blue-stained gel showing composition of the purified PRC2 complex.

b. RT-qPCR analysis showing relative expression of *NEGR1*, *ACPL2*, *IL6ST1*, and *HS2ST1* in Ctrlsi, *MEG3*si (*MEG3*si2 and *MEG3*si3), and *EZH2* siRNA-transfected BT-549 cells (± SD, n = 3). *MEG3*si3-treated cells were used for gene expression analysis using microarray and RNA-seq whereas *MEG3*si2 was an alternative *MEG3* siRNA used for validation of findings from *MEG3*si3.

c. RT-qPCR analysis showing downregulation of *MEG3* in Ctrlsi-, *MEG3*si2-, and *MEG3*si3-transfected BT-549 cells.

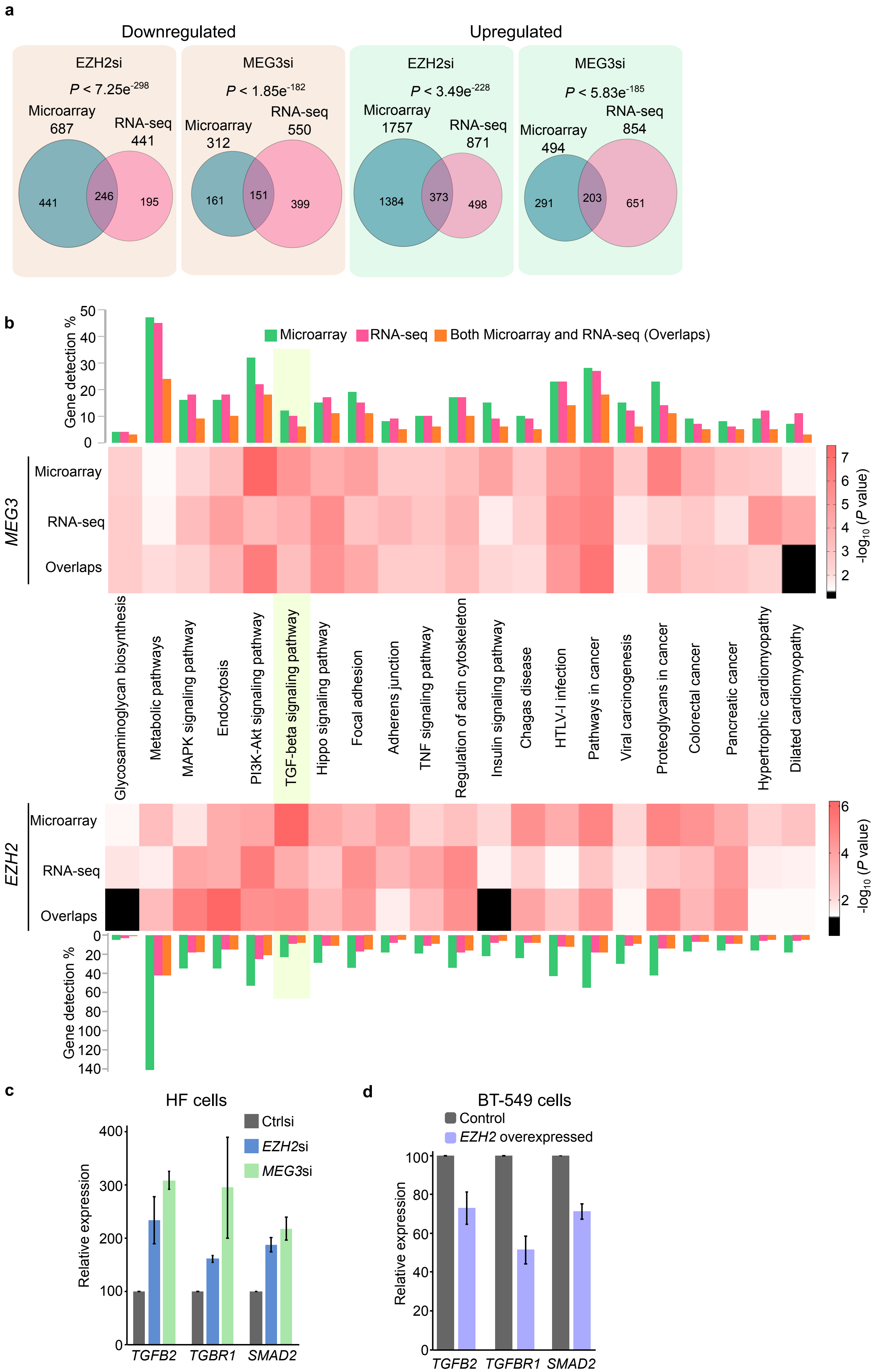

a
Downregulated
Upregulated
EZH2si
P < 7.25e-298
Microarray 687
RNA-seq 441
441
246
195
MEG3si
P < 1.85e-182
Microarray 312
RNA-seq 550
161
151
399
EZH2si
P < 3.49e-228
Microarray 1757
RNA-seq 871
1384
373
498
MEG3si
P < 5.83e-185
Microarray 494
RNA-seq 854
291
203
651
b
Microarray
RNA-seq
Both Microarray and RNA-seq (Overlaps)
Gene detection %
MEG3
Microarray
RNA-seq
Overlaps
-log10 (P value)
Glycosaminoglycan biosynthesis
Metabolic pathways
MAPK signaling pathway
Endocytosis
PI3K-Akt signaling pathway
TGF-beta signaling pathway
Hippo signaling pathway
Focal adhesion
Adherens junction
TNF signaling pathway
Regulation of actin cytoskeleton
Insulin signaling pathway
Chagas disease
HTLV-I infection
Pathways in cancer
Viral carcinogenesis
Proteoglycans in cancer
Colorectal cancer
Pancreatic cancer
Hypertrophic cardiomyopathy
Dilated cardiomyopathy
EZH2
Microarray
RNA-seq
Overlaps
-log10 (P value)
Gene detection %
c
HF cells
Ctrlsi
EZH2si
MEG3si
Relative expression
TGFB2
TGBR1
SMAD2
d
BT-549 cells
Control
EZH2 overexpressed
Relative expression
TGFB2
TGFBR1
SMAD2

Supplementary Figure 4. Data related to RNA-seq and RT-qPCR validation.

a. Figure showing the overlap of the deregulated protein-coding genes identified by microarray and RNA sequencing after downregulation of *MEG3* and *EZH2* using siRNA in BT-549 cells. The *P* values were obtained by performing a hypergeometric test using all protein-coding genes as a background.

b. Pathway analysis of the overlapped genes from microarray and RNA-seq after *MEG3* and *EZH2* downregulation in BT-549 cells, using KEGG annotation.

c. RT-qPCR analysis of *TGFB2*, *TGFBR1*, and *SMAD2* gene expression in Ctrlsi-, *MEG3*si-, and *EZH2*si-transfected HF cells (± SD, n = 3).

d. RT-qPCR analysis of *TGFB2*, *TGFBR1*, and *SMAD2* gene expression after overexpression of *EZH2* in BT-549 cells, compared to control cells, 48 hours after transfection.

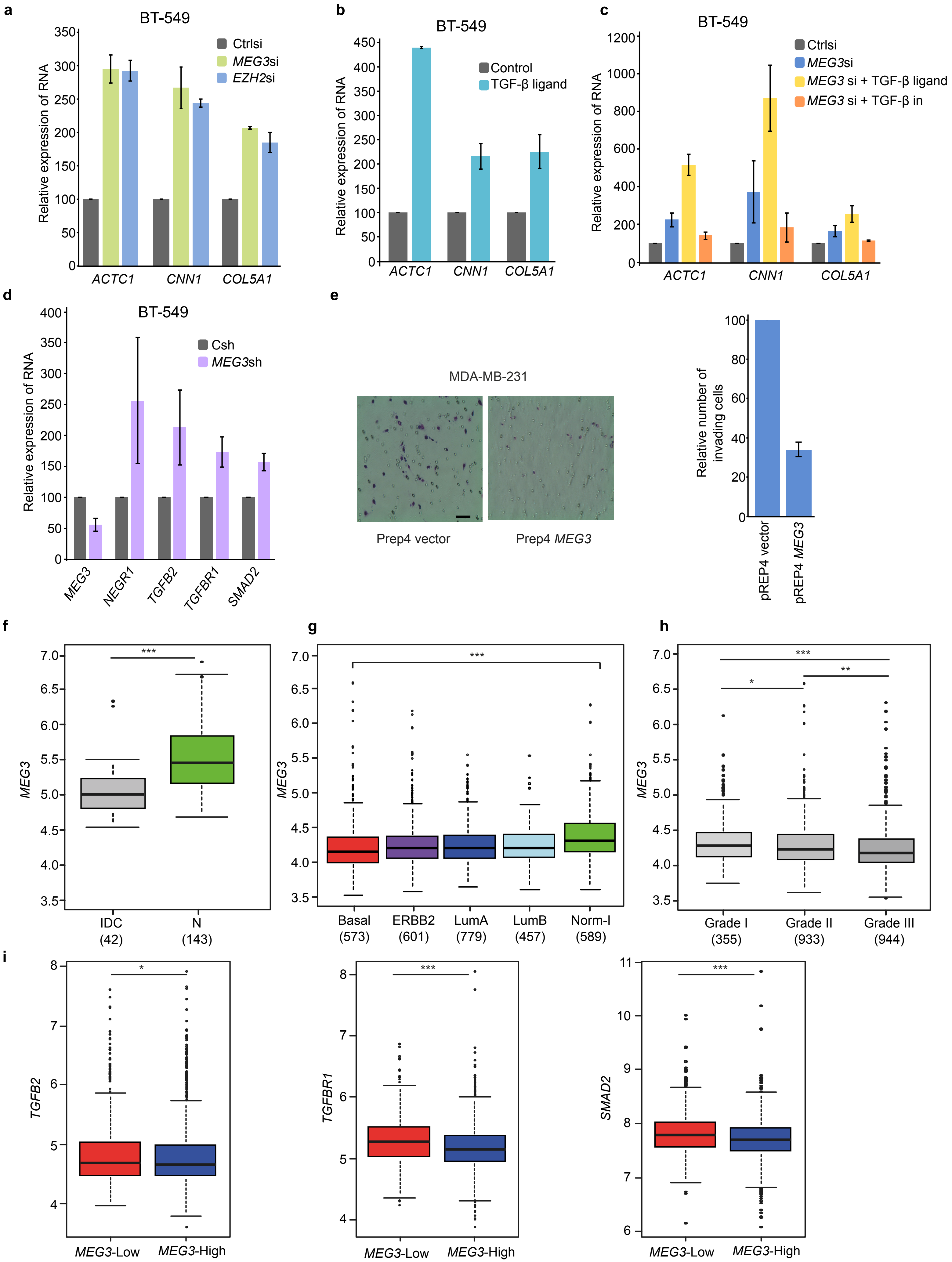

a
BT-549
Relative expression of RNA
Ctrlsi
MEG3si
EZH2si
ACTC1
CNN1
COL5A1
b
BT-549
Control
TGF-β ligand
c
BT-549
Ctrlsi
MEG3si
MEG3 si + TGF-β ligand
MEG3 si + TGF-β in
d
BT-549
Csh
MEG3sh
MEG3
NEGR1
TGFB2
TGFBR1
SMAD2
e
MDA-MB-231
Prep4 vector
Prep4 MEG3
Relative number of invading cells
pREP4 vector
pREP4 MEG3
f
MEG3
IDC (42)
N (143)
g
Basal (573)
ERBB2 (601)
LumA (779)
LumB (457)
Norm-l (589)
h
Grade I (355)
Grade II (933)
Grade III (944)
i
TGFB2
TGFBR1
SMAD2
MEG3-Low
MEG3-High

Supplementary Figure 5. *MEG3* regulates *TGF-β* pathway genes.

a. RT-qPCR analysis of *ACTC1*, *CNN1*, and *COL5A1* gene expression in Ctrlsi-, *MEG3*si-, and *EZH2*si-transfected BT-549 cells (± SD, n = 3).

b. RT-qPCR analysis of *ACTC1*, *CNN1*, and *COL5A1* gene expression in BT-549 cells after incubation with TGF-β2 ligand.

c. RT-qPCR analysis of *ACTC1*, *CNN1*, and *COL5A1* gene expression in BT-549 cells transfected with Ctrlsi and Ctrlsi transfection followed by incubation with TGF-β2 ligand (Ctrlsi+ TGF-β ligand). Expression was also measured in *MEG3*si and *MEG3*si transfection followed by incubation with TGF-β inhibitor (*MEG3*sh+*TGF-β*in). The bar graph shows relative quantification of expression (± SD, n = 3) compared to the Ctrlsi transfection.

d. RT-qPCR analysis of *MEG3*, *NEGR1*, *TGFB2*, *TGFBR1*, and *SMAD2* gene expression in Ctrlsh, *MEG3*sh-expressing BT-549 cells (± SD, n = 3).

e. Images depicting the matrigel invasion of MDA-MB-231 cells after overexpression of *MEG3*. A bar graph next to the images shows quantification (± SD, n = 3) of the matrix invaded MDA-MB-231 cells upon overexpression of *MEG3* relative to the vector control. Scale bar represents 5 μm.

f–h. *MEG3* lncRNA expression is downregulated in high-grade breast tumors.
f. *MEG3* is expressed at a significantly lower level in invasive ductal carcinoma than in normal breast tissue. The y-axis is a $\log_2$ intensity scale of gene expression from published microarray dataset GSE10780 (Chen et al. 2010).
g. *MEG3* expression is lowest in the most aggressive 'basal' molecular subtype of breast cancer and highest in the 'normal-like' subtype.
h. High-grade breast cancer subtypes show lower *MEG3* expression. IDC, invasive ductal carcinoma; N, normal breast tissue; Lum, luminal; Norm-l, normal-like; ERBB2, human epidermal growth factor receptor 2 positive, estrogen receptor alpha negative subtype. The y-axes in panels are a $\log_2$ intensity scale of batch-corrected RNA expression levels from a compendium of 17 Affymetrix primary breast cancer datasets. Wilcoxon rank sum test *$P < 0.05$, **$P < 0.001$, ***$P < 0.0001$.

i. Expression of *TGF-β* genes *TGFB2*, *TGFBR1*, and *SMAD2* in *MEG3*-low and *MEG3*-high primary tumors (separated at lower quartile). The *P* values were calculated using Student's *t*-test. *$P < 0.05$, **$P < 0.0001$.

a

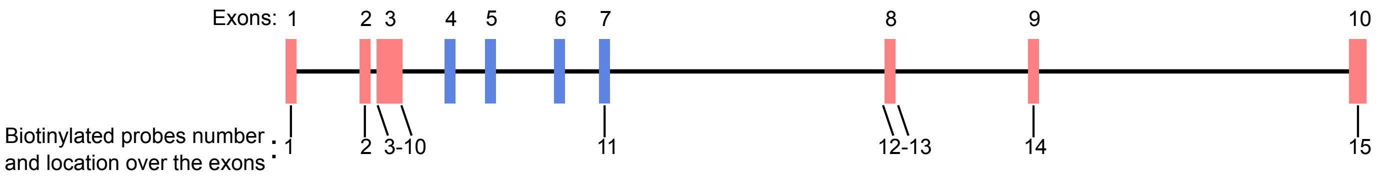

b

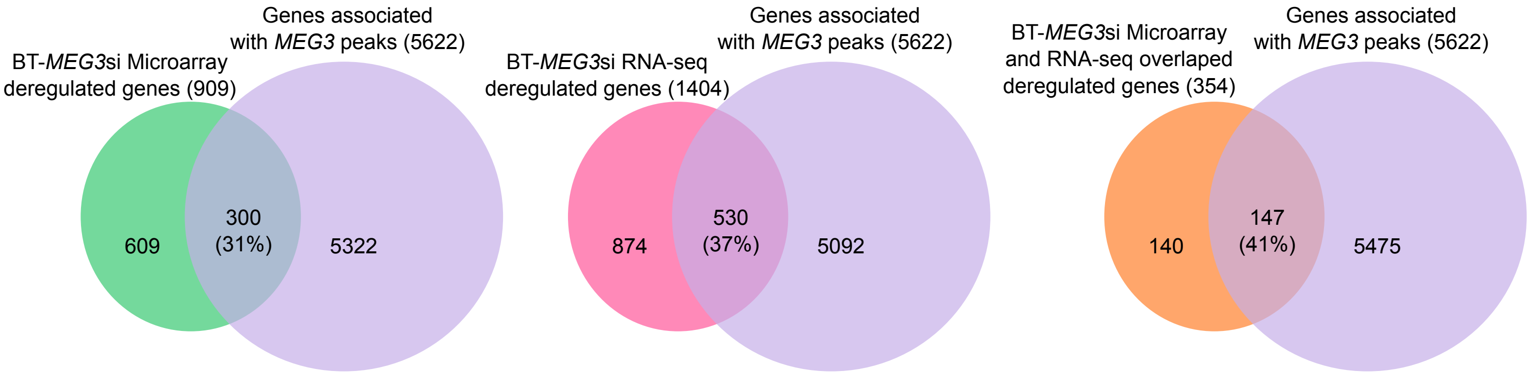

c

Deregulated genes	Genes associated with *MEG3* peaks		Number of deregulated genes with distal peaks having H3K4me1 signal
	Promoter	Distal	
Microarray	8	292	52 ($P < 2.27e^{-24}$)
RNA-seq	13	517	110 ($P < 2.34e^{-58}$)
Both Microarray & RNAseq	5	142	31 ($P < 4.65e^{-18}$)

Supplementary Figure 6. *MEG3* gene structure, association of the *MEG3* peaks to the deregulated genes from microarray, RNA-seq and the overlapped deregulated genes from microarray and RNA-seq.

a. Schematic diagram showing the exon structure of the *MEG3* gene along with the location of the antisense probes used in the ChOP pulldown. The exons shown in red are constitutively expressed while the exons shown in blue are alternatively spliced. The numbering at the top corresponds to the exon number. The numbering below the diagram corresponds to 15 antisense probes used in the ChOP assay. Of the 15 probes, 14 were complementary to the constitutively expressed exons (red).

b. Venn-diagrams showing the extent overlap (indicated in percentage) between genes associated with *MEG3* peaks and the deregulated genes from microarray (left panel), RNA-seq (middle panel) and the overlapped deregulated genes from microarray and RNA-seq (right panel).

c. Table shows the deregulated genes having *MEG3* peaks at the promoter and distal regulatory regions. Also, it shows *MEG3* peaks having H3K4me1 signals. The *P* values were obtained by performing a hypergeometric test using all protein-coding genes as a background.

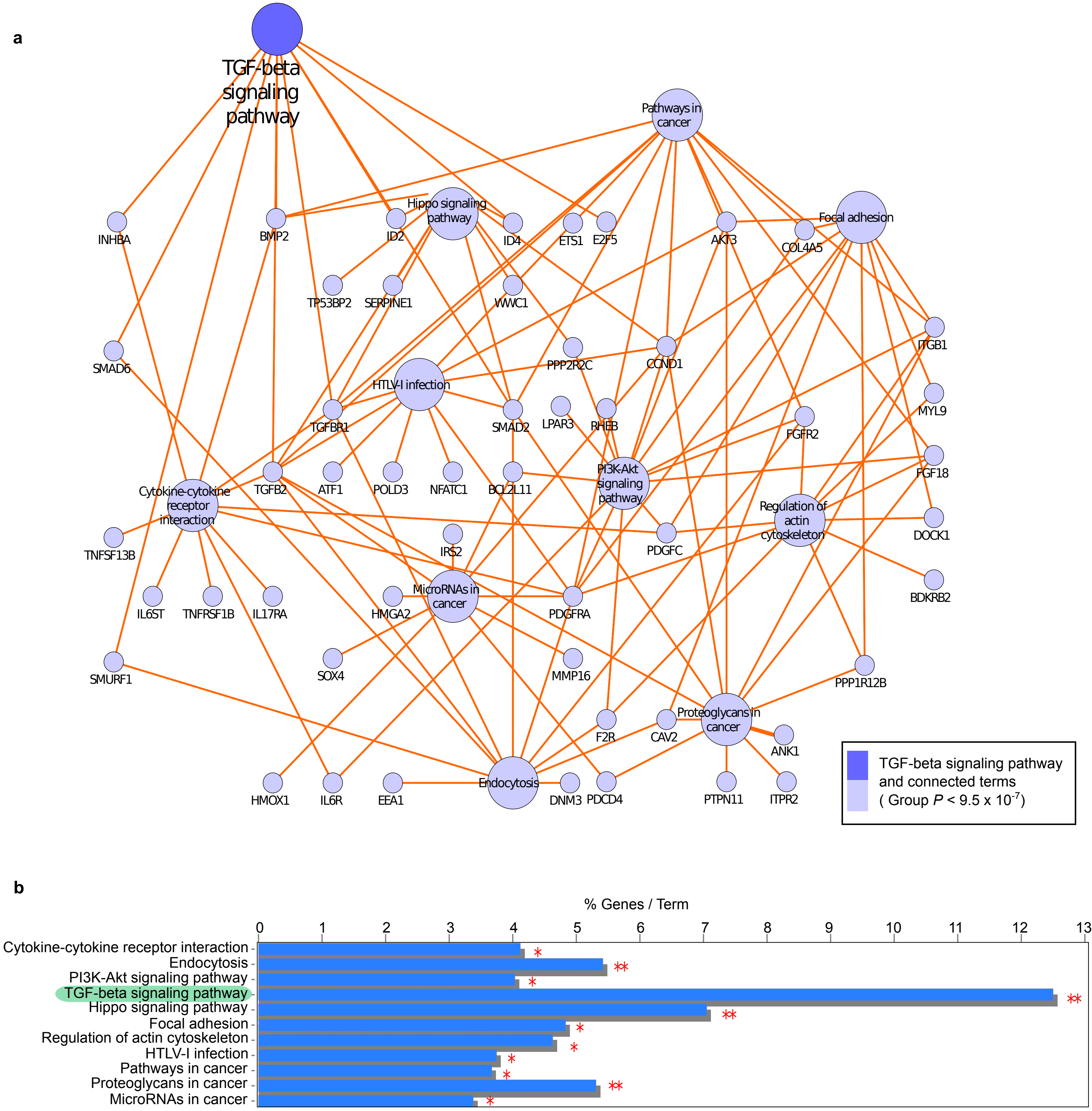

Supplementary Figure 7. Network of genes and pathways.

a. Network of pathways showing the *TGF-β* pathway as a major term based on 300 *MEG3* peak-associated deregulated genes after *MEG3*si transfection of BT-549 cells.

b. The *TGF-β* pathway has a significant number of genes among the networks. Asterisks in red denote statistical significance.

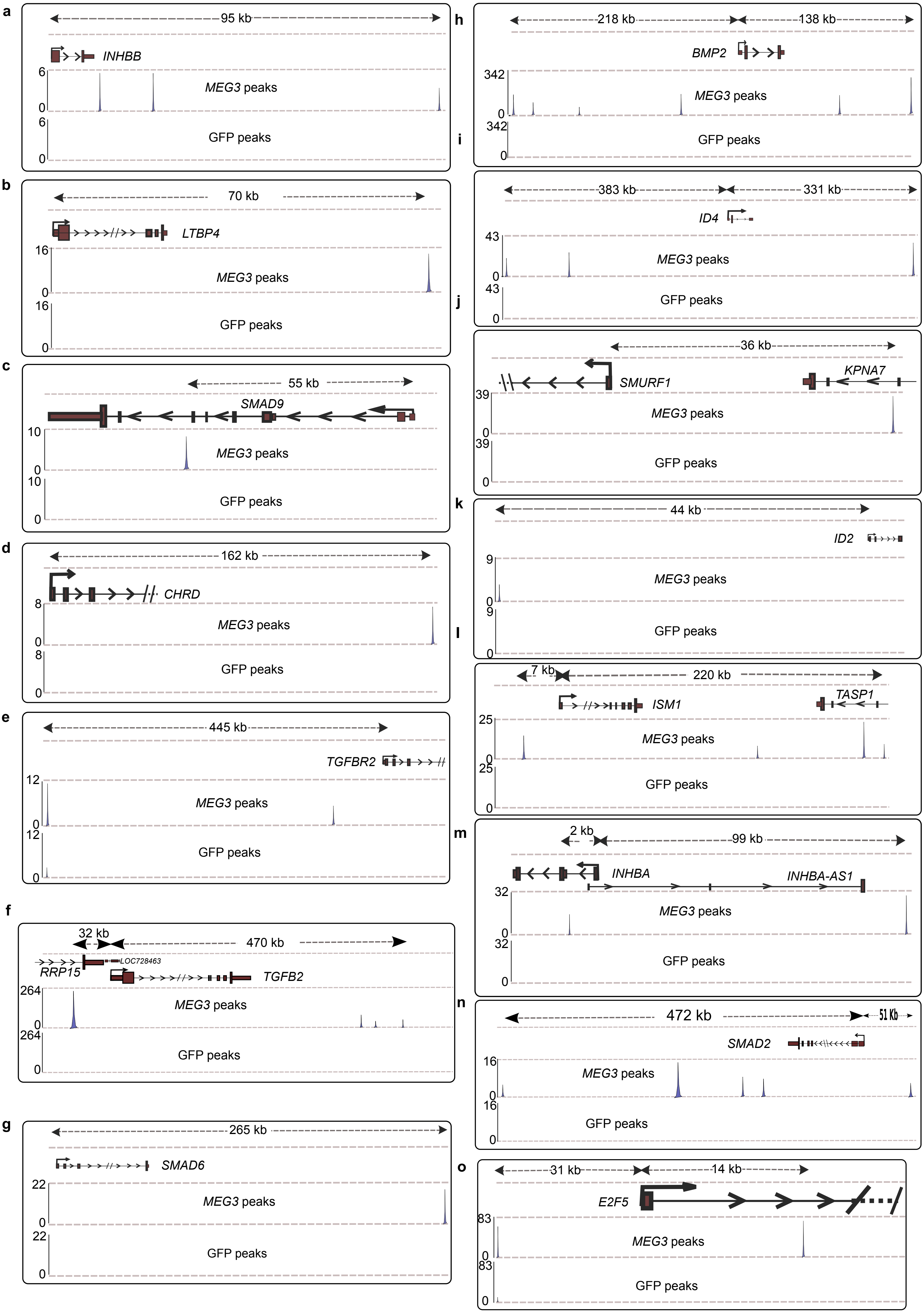

a
95 kb
INHBB
6
0
MEG3 peaks
6
0
GFP peaks
b
70 kb
LTBP4
16
0
MEG3 peaks
16
0
GFP peaks
c
55 kb
SMAD9
10
0
MEG3 peaks
10
0
GFP peaks
d
162 kb
CHRD
8
0
MEG3 peaks
8
0
GFP peaks
e
445 kb
TGFBR2
12
0
MEG3 peaks
12
0
GFP peaks
f
32 kb
470 kb
LOC728463
RRP15
TGFB2
264
0
MEG3 peaks
264
0
GFP peaks
g
265 kb
SMAD6
22
0
MEG3 peaks
22
0
GFP peaks
h
218 kb
138 kb
BMP2
342
0
MEG3 peaks
i
342
0
GFP peaks
383 kb
331 kb
ID4
43
0
MEG3 peaks
j
43
0
GFP peaks
36 kb
SMURF1
KPNA7
39
0
MEG3 peaks
39
0
GFP peaks
k
44 kb
ID2
9
0
MEG3 peaks
l
9
0
GFP peaks
7 kb
220 kb
ISM1
TASP1
25
0
MEG3 peaks
25
0
GFP peaks
m
2 kb
99 kb
INHBA
INHBA-AS1
32
0
MEG3 peaks
32
0
GFP peaks
n
472 kb
51 Kb
SMAD2
16
0
MEG3 peaks
16
0
GFP peaks
o
31 kb
14 kb
E2F5
83
0
MEG3 peaks
83
0
GFP peaks

Supplementary Figure 8. *MEG3* peaks associated to the *TGF-β* pathway genes.

a–o. Location of the *MEG3* peaks associated with the *TGF-β* pathway genes relative to the transcription start site. The scale on the y-axis shows ChOP-seq intensities in log10 scale.

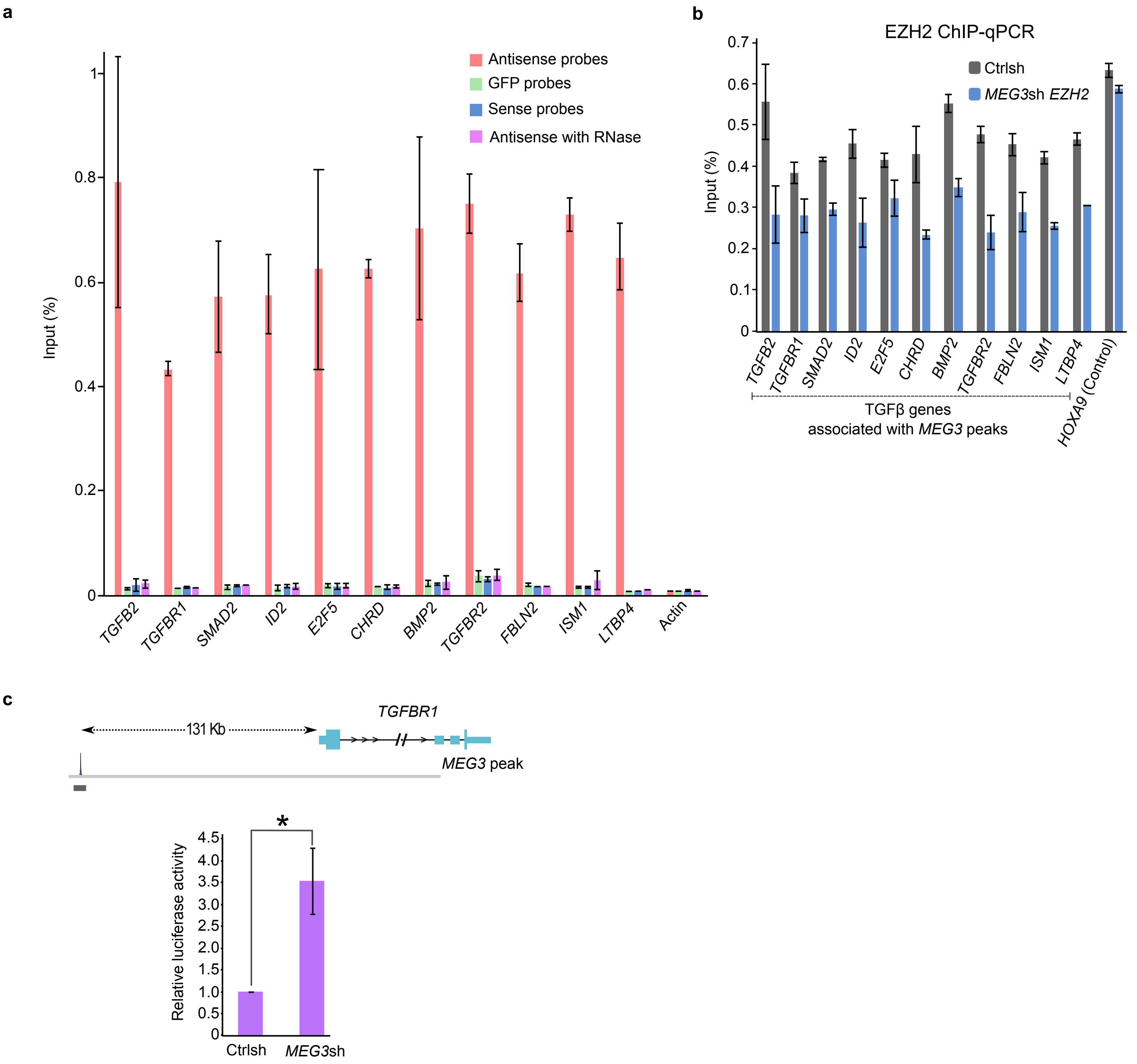

Supplementary Figure 9. ChOP-qPCR validation.

a. ChOP-qPCR validation of the *MEG3* binding sites associated with the *TGF-β* pathway genes (± SD, n = 3). We detected enrichment of the *MEG3* peak sequences only when ChOP was performed with antisense oligos. Pulldown with sense oligos or with non-specific GFP oligos did not show any enrichment. RNase A treatment of the sonicated chromatin prior to ChOP pulldown resulted in complete loss of enrichment. β-actin was used as a negative control.

b. ChIP-qPCR result showing enrichment (percentage of input) of EZH2 over the *MEG3* peaks associated with the *TGF-β* genes in Ctrlsh and *MEG3*sh cells. (± SD, n = 3).

c. Enhancer activity of the *TGFBR1*-associated *MEG3* peak. The bar graph represents the relative normalized firefly luciferase activity of the DNA element (around 1,200 bp) containing *MEG3* peak associated with the *TGFBR1* in Ctrlsh and *MEG3*sh transduced cells. Firefly luciferase activity was normalized with *Renilla* luciferase and is presented as luciferase activity in MEG3sh cells relative to Ctrlsh cells (± SD, n = 3). The *P* value was calculated using Student's *t*-test. The schematic diagram above the graph shows the location and distance of the *MEG3* peak from the *TGFBR1* gene promoter. The small gray bar below the *MEG3* peak indicates the cloned DNA fragment.

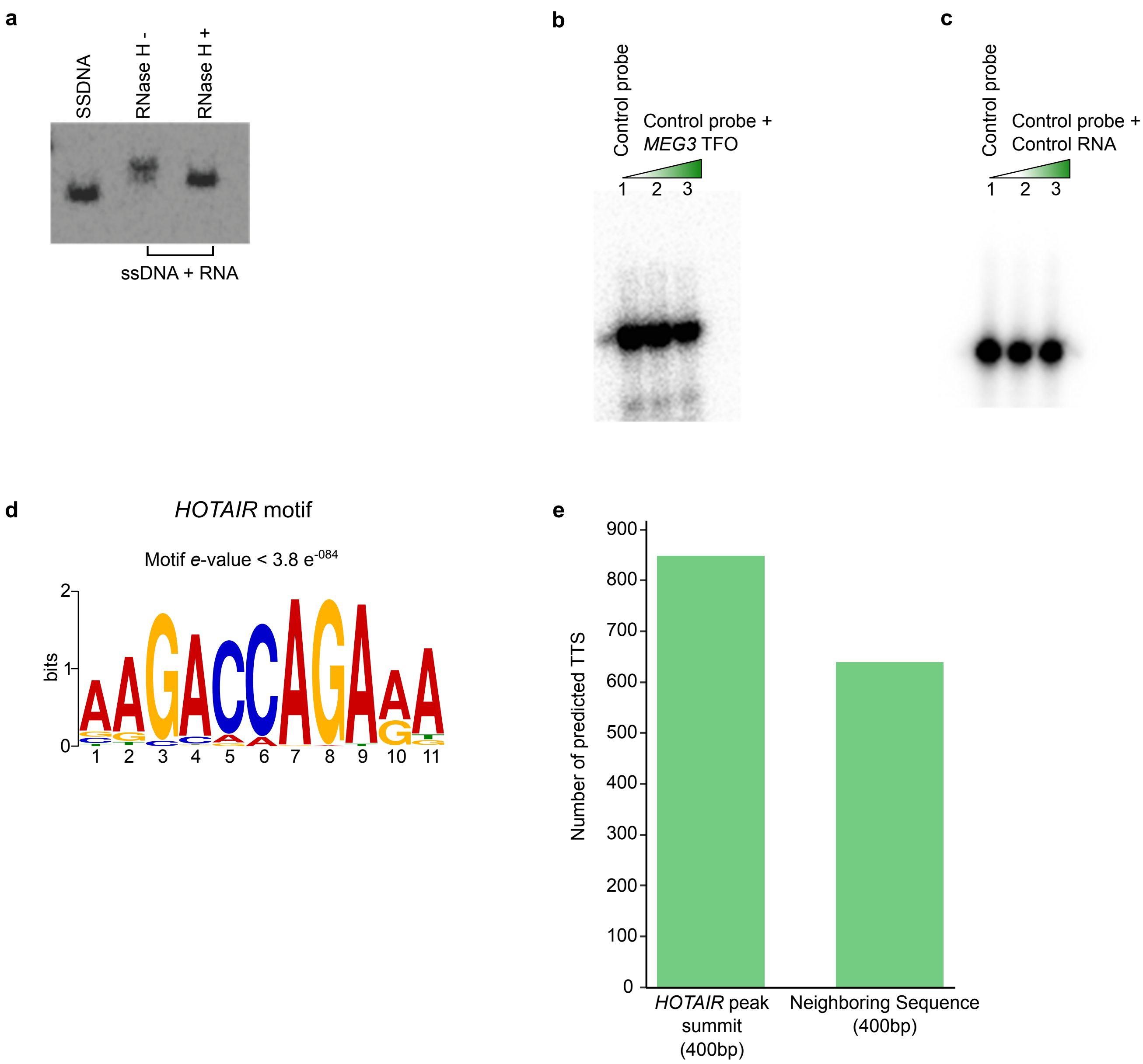

Supplementary Figure 10. Data related to *in vitro* triplex assay.

a. RNase H digestion control. To the far left is the ^{32}P-labeled ssDNA probe. The middle lane corresponds to the DNA-RNA hybrid (^{32}P-labeled ssDNA probe + ssRNA oligo). The right lane corresponds to the DNA-RNA hybrid treated with RNase H.

b, c. Electrophoretic mobility shift assay.
b. End-labeled control dsDNA oligo was incubated alone (lane 1) or with increasing concentrations (1.0 μM, 2.0 μM) of *MEG3* ssRNA TFO (lanes 2 and 3).
c. End-labeled control dsDNA oligo was incubated alone (lane 1) or with increasing concentrations (1.0 μM, 2.0 μM) of control ssRNA oligo (lanes 2 and 3).

d. GA-rich motifs are enriched over the *HOTAIR* binding sites. *HOTAIR* binding sites were obtained from Chu. C et al. (2011) [3].

e. The number of triplex target sites (TrTS) over the *HOTAIR* peak summits and their neighboring regions, as predicted by Triplexator.

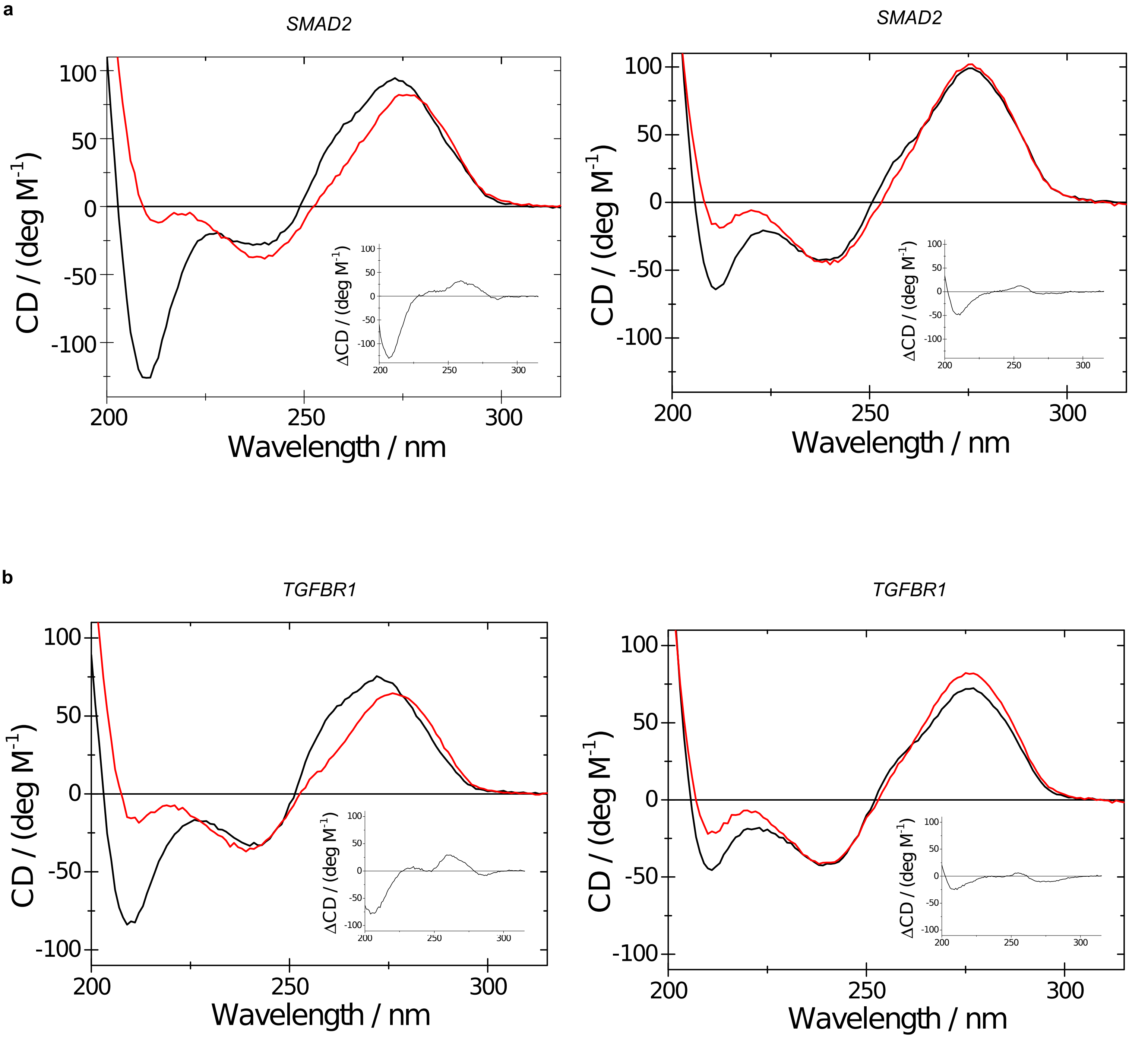

Supplementary Figure 11. Data related to CD spectra .

a. Left panel: CD spectra of a 1:1 mixture of *SMAD2* dsDNA and *MEG3* TFO (ssRNA), shown in black, and of a 1:1 mixture of *SMAD2* dsDNA and control ssRNA, shown in red. Inset: The difference between the two spectra. Right panel: The sum of the individual CD spectra for *SMAD2* dsDNA and *MEG3* TFO (ssRNA), shown in black, and the sum of the individual CD spectra for *SMAD2* dsDNA and control ssRNA, shown in red. Inset: the difference between the two spectra.

b. Left panel: CD spectra of a 1:1 mixture of *TGFBR1* dsDNA and *MEG3* TFO (ssRNA), shown in black, and a 1:1 mixture of *TGFBR1* dsDNA and control ssRNA, shown in red. Inset: The difference between the two spectra. Right panel: The sum of the individual CD spectra for *TGFBR1* dsDNA and *MEG3* TFO (ssRNA), shown in black, and the sum of the individual CD spectra for *TGFBR1* dsDNA and control ssRNA, shown in red. Inset: the difference between the two spectra.

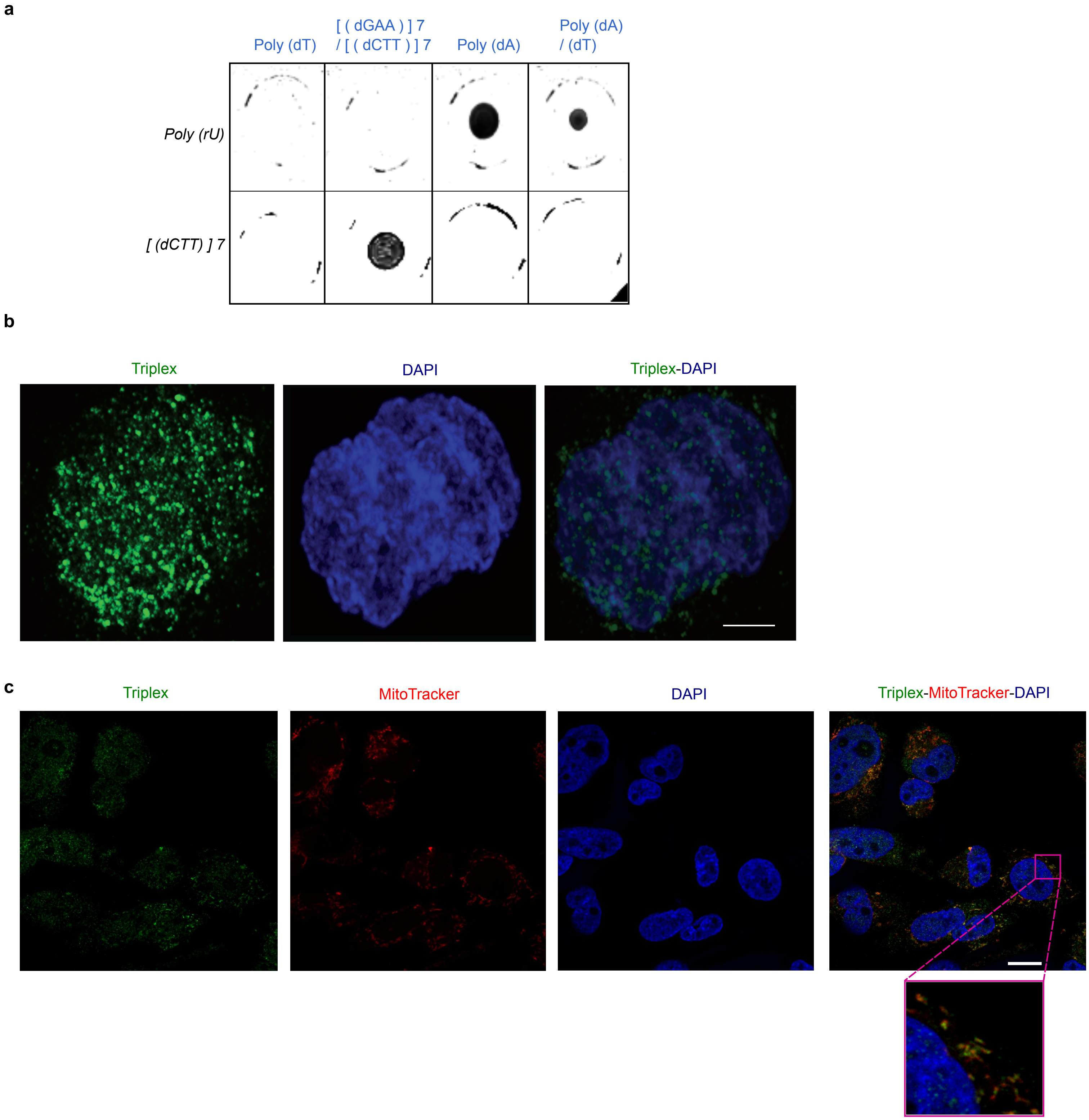

Supplementary Figure 12. Anti-triplex dA.2rU antibody validation and triplex staning.

a. DNA sequences (specified in blue) were fixed on membranes and allowed to react with solutions of either RNA homopolymer or non-homopolymeric oligo-deoxyribonucleotide (italicized). Triplex immunodetection was carried out with anti-(dA)/2(rU) followed by color development reaction (dark spots).

b. High-resolution confocal microscopic images of triplex structures in BT-549 cells. Triplex structures (green, left panel), nuclear staining with DAPI (blue, center panel), and overlay of triplex signals with DAPI (right panel). Scale bar represents 0.5 μm.

c. Confocal microscopic images showing overlap of the cytoplasmic triplex structures and mitochondrial staining in BT-549 cells. Mitochondria were labeled with MitoTracker (red) and triplex structures were detected with anti-triplex dA.2rU antibody (Green) and nucleus was stained with DAPI. The image in the extreme right represents the overlap of the mitochondrial and triplex staining along with DAPI. The zoom in image represents the the co-localization of the red (mitochondrial staining) and green (triplex staining) signals. Scale bar represents 5 μm.

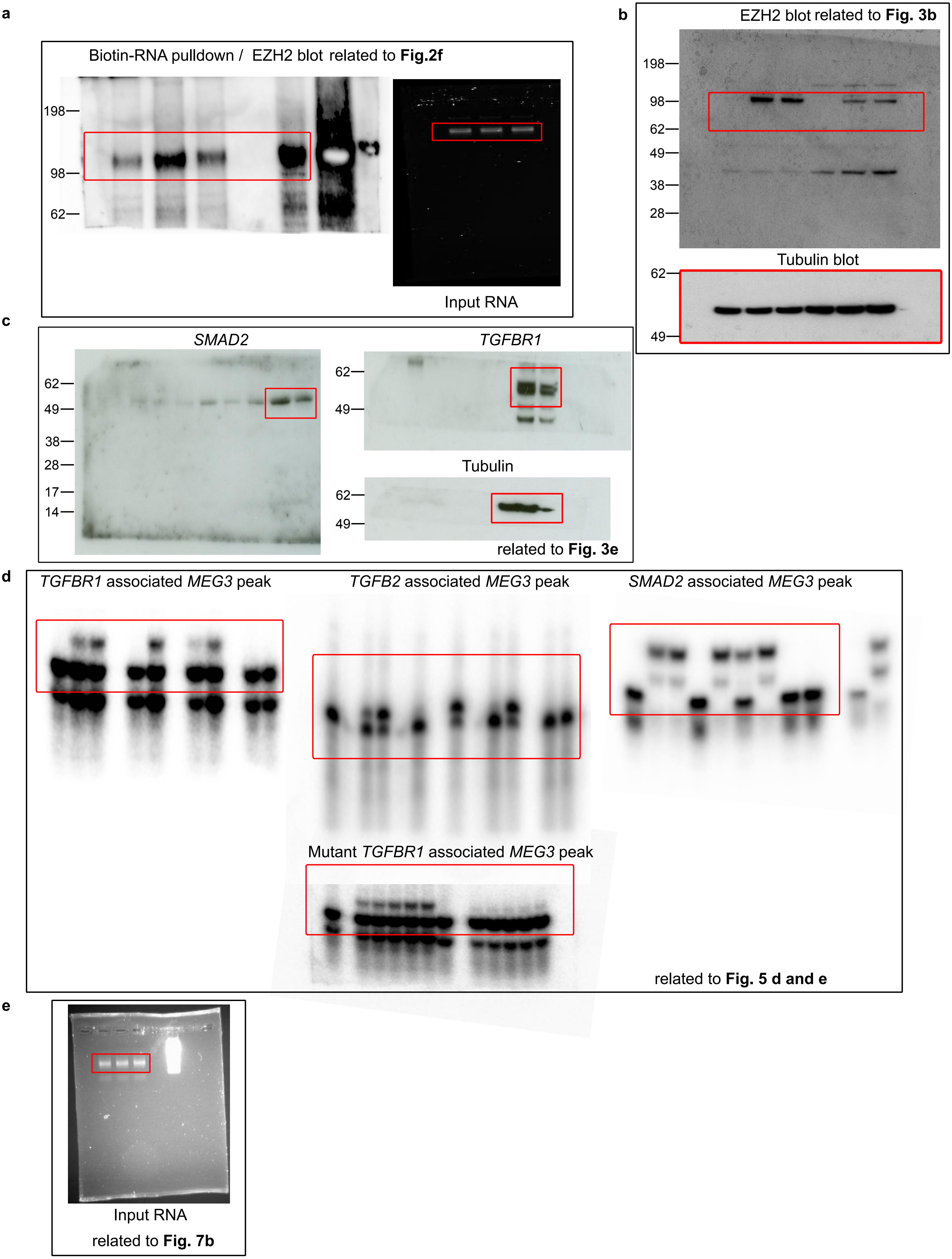

Supplementary Figure 13. Uncut blots and gels. The red boxed regions are presented in the manuscript.

Supplementary Methods

Chromatin RNA immunoprecipitation followed by high throughput sequencing (ChRIP-seq)

Chromatin RNA immunoprecipitation (ChRIP) was performed using BT-549 cells adapting the protocols from Mondal *et al.*[4] and Kuo *et al.*[5] with the modifications as detailed below. BT-549 cells were plated on tissue culture dish (7-8 million cells/150mm plate) and incubated overnight (14-16 hours) with 4-thiouridine (4sU, Sigma) at a final concentration of 100 µM in cell culture media. Next day, Actinomycin D (ActD, 5µg/ML) was added to the media and incubated for 40-45 mins. To check the efficacy of ActD treatment, cells were incubated with and without ActD and RNA was extracted and assayed level of *c-Myc* RNA, with a short half-life, by RT-qPCR. After ActD incubation, cells were washed 2 times with PBS and crosslinked with 1% formaldehyde for 10 minutes with gentle shaking. Crosslinking was stopped by adding glycine to a final concentration of 125mM and incubated for 5 minutes with gentle shacking. Cells were subsequently washed twice with PBS followed by crosslinking on ice with UV. Cells were removed by scraping from the plate and resuspended in cold PBS. Nuclei were isolated using 1X nuclei isolation buffer (40mM Tris-HCl pH7.5, 20mM MgCl2, 4% tritonX-100, 1.28M sucrose) and washed again with PBS. The isolated crosslinked nuclei were resuspended in lysis buffer (0.1%SDS, 0.5% TritonX 100, 20mM Tris-HCl pH7.5, 150mM NaCl, 1ml lysis buffer per 10 million of cells) supplemented with RNasein (Promega) and subjected to sonication (Bioruptor, 20-30 cycles) to obtain chromatin fragments of about 1 kb. 50-60 µg of soluble chromatin was used in each chromatin immunoprecipitation and incubated with 5 µg of anti-EZH2 (Active motif) and anti-H3K27me3 (Merck-Millipore) antibody. Antibody bound chromatin was washed according to our earlier published protocol with buffers were supplemented with RNasein. Protein A magnetic beads bound to immunoprecipitated chromatin were resuspended in 10X volume of elution buffer (100mM NaCl, 10mM Tris 7.5, 1mM EDTA, 0.5% SDS) containing proteinase K. proteinase K treatment was carried out at 55°C for 45 minutes followed by heating at 95°C for 10 minutes to reverse crosslinking. Chromatin bound RNA was extracted with Trizol (Life Technologies) and subjected to DNase I (Promega) treatment to remove traces of DNA. Since the chromatin bound RNA yield from one single experiment is suboptimal for high-throughput sequencing, we pooled RNA from 6 to 8 ChRIP pulldowns and the sequencing library was made using SOLiD Total RNA-Seq Kit and sequenced the library using SOLiD platform (Applied Biosystem). SOLiD Total RNA-Seq Kit allows obtaining strand-specific

RNA sequencing information. Since library preparation in SOLID protocol involves ligation of RNA adaptors to the RNA fragments by RNA ligase, no contamination from DNA fragments is expected. For input, nuclear RNA was isolated from 4sU and ActD treated BT-549 cells and depleted ribosomal RNA using RiboMinus™ Eukaryote System v2 (Life Technologies). For ChRIP validation, we followed the same protocol as described above with or without ActD treatment using antibodies against H3K27me3, EZH2, H3K4me2 (Merck-Millipore) and non-specific Rabbit IgG (Merck-Millipore).

RNA Sequencing and alignment of H3K27me3, EZH2 and nuclear RNA samples

Using standard Applied Biosystems bioinformatics pipeline adapter sequences were removed from EZH2, H3K27me3 and nuclear RNA (Input) raw sequencing reads. We then aligned clean raw sequencing reads (25-75 nucleotides long) to the reference hg19 genome using Lifescope 2.5 [http://www.lifetechnologies.com/lifescope], splice junction-aware aligner from Applied Biosystems with default parameters (number of mismatches allowed: 2). The alignment files were further processed for quality filtering (mapping quality, MAPQ >=30; maximum mapping quality in SOLID, MAPQ=50) using SAMtools [6] to avoid multiple mapped reads and obtained 29841706, 4384490, 8084998 mappable reads in EZH2, H3K27me3 and nuclear RNA (Input) samples, respectively.

Transcriptome reconstruction using nuclear RNA sample

Transcriptome reconstruction was performed by Cufflinks [7] on nuclear RNA (Input) sample using GENCODE v11 annotation [8]. For non-annotated transcript discovery we used guided mode (parameter: --*GTF-guide*) in Cufflinks which uses RABT (Reference Annotation Based Transcript) assembly [9]. Transcripts were assembled with standard SOLiD library type parameter of *fr-secondstrand* for strandedness and the transfags are merged if the distance between them is less than 100 bp (parameter: --*overlap-radius*). The assembled reads were also multi-read corrected during Cufflinks procedure. Later the non-annotated transcripts which overlap with exons of known transcripts (GENCODE v11) were removed by retaining intronic and intergenic non-annotated transcripts.

Transcript abundance, differential expression analysis, finding T to C conversion and peak calling

The predicted known and non-annotated transcripts (13,957 lncRNAs) from Cufflinks using nuclear RNA sample were used as a transcript reference (Instead of standard GENCODE

reference) to find corresponding gene expression levels in input, EZH2 and H3K27me3 samples. Using HTSeq [http://www.huber.embl.de/users/anders/HTSeq/doc/overview.html] we have quantified gene expression levels (number of reads over each transcript) from all samples. The differential enrichment of transcripts in EZH2 and H3K27me3 ChRIP sequencing data were determined by using input as control for both the samples. The R package, edgeR no-replicate mode [10] was used on gene expression data obtained from HTSeq to find significantly enriched lncRNAs in EZH2 and H3K27me3 ChRIP over input (fold change over Input >= 2). We obtained 276 lncRNAs enriched in both EZH2 and H3K27me3 ChRIP (hypergeometric *P* value was used to calculate significance of the overlap, 6,205 lncRNAs were used as background) [11]. To visualize the differences in distribution of the reads in EZH2, H3K27me3 and input samples we have used MACS peak caller [12]. Some of the enriched non-annotated transcripts were visualized with phastCons scores, conservation across 46 vertebrate species using Multiz genome-wide alignments [13] and RNA-seq coverage (predicted transcript regions) for 10 different tissues from Illumina's Human BodyMap 2.0 project [ArrayExpress: Body Map 2.0, query ID: E-MTAB-513]. For repressive chromatin enriched non-annotated transcripts we have checked for probable coding potential using Coding Potential Assessment Tool (CPAT) [14].

We have used Freebayes 0.6.3 (Bayesian genetic variant detector) [15] to identify conversion sites in our ChRIP RNA-seq samples and looked for T to C (Thymine to Cytosine) transitions. We obtained significantly higher number of T to C changes compared to other transitions. The transitions (T to C) were considered only if the minimum read depth >= 2 (total number of reads covered per transition position) [16]. The filtered T to C transitions were mapped to the known lncRNAs and non-annotated transcripts identified by Cufflinks.

Expression analysis by microarray and RNA sequencing after siRNA transfection

Total RNA was extracted 48 hours post siRNA transfection. Microarray hybridization was performed using Human Genome U133A 2.0 Array and the data was processed by following standard protocols from Uppsala Affymetrix Array Platform. The probe sets from *EZH2*si and *MEG3*si microarray samples were selected on the basis of presence of signal and fold change compared to si*CTRL*. A transcript is considered deregulated if it has fold change ≥2 (up-regulated) ≤ -2 (down-regulated) for individual siRNA treated samples. We have performed hypergeometric test on the overlaps of the deregulated genes in *MEG3*si and *EZH2*si using 30,000 genes as a background [17].

RNA-sequencing was performed using Illumina platform, Beijing Genomics Institute (BGI). Sample RNA libraries were prepared from ribosomal RNA depleted total RNA as per the standard Illumina library preparation protocol and the library products (*Ctrl*si, *EZH2*si and *MEG3*si) were sequenced using Illumina HiSeq™ 2000. The obtained raw reads were cleaned by removing adapter sequences and the cleaned reads having base quality of ≤ 5 in greater than 50 % of bases were considered for the alignment to the reference genome hg19 using SOAP aligner [18] by only allowing 2 mismatches. We obtained 43,297,701 reads for *Ctrl*si, 53,405,443 reads for *EZH2*si and 40,502,364 reads for *MEG3*si. Adapter removal, quality control and differential expression analysis was performed as per standard BGI bioinformatics analysis pipeline [http://bgitechsolutions.com/service-solutions/services/transcriptomics/rna-seq-quantification/].

RNA Immuno-precipitation (RIP), PRC2-*MEG3* binding assay and Biotin-RNA pull-down assay

RIP assay was carried out using protocol from Guttman *et al.* [19] with minor modifications. BT-549 cells were incubated for 16 hours with photo-reactive 4-Thiouridine (4SU) and expose to UV treatment to cross-link RNA-protein interactions followed by cell lysis, immunoprecipitation, bead washing and RNA isolation after pull-down.

RIP was performed following transfection of 1 µg of WT *MEG3*, Δ340-348 *MEG3 and* Δ345-348 *MEG3* constructs into BT-549 cells using lipofectamine 2000 reagent (Life Technologies). To distinguish the endogenous *MEG3* from the ectopically expressed *MEG3*, we designed RT-qPCRs primers in such a way that one of the RT-qPCR primers map to the transcribed vector portion and the other to *MEG3* RNA. Endogenous *MEG3* was used as a RIP positive control and *U1SnRNA* as a negative control.

PRC2-*MEG3* binding assay was carried out using commercially purchased PRC2 protein complex (Active motif) and *in vitro* synthesized wild-type (WT) or mutant *MEG3* RNAs in RNA binding buffer (20 mM Tris-HCl (pH 7.5), 180 mM NaCl, 0.1% Nonidet P-40, 1.5 mM MgCl2, 13.2% glycerol) for 20min at 25°C. PRC2-*MEG3* complex was captured by EZH2 antibody (Active motif) coupled to protein A beads, washed 4 times in rotation to get rid of non-bound *MEG3* RNAs. PRC2 bound *MEG3* RNA was extracted from the beads using Trizol (life technologies). As a negative control, a reaction without PRC2 protein was used which helps to detect background non-specific binding of RNA to the antibody and beads.

Trizol extracted RNA was used in RT-qPCR to detect the enrichment of the bound *MEG3* RNA over the negative control.

Biotin-RNA pull-down assay was carried out as in Tsai *et al.* [20] with the modifications as followed. Biotin-labeled WT sense, antisense and Δ345-348 *MEG3* RNAs were synthesized using biotin RNA labeling mix (Roche) and incubated the *in vitro* synthesized RNAs with nuclear lysate from BT-549 cells followed by capturing with streptavidin-magnetic beads. Bound proteins were eluted in SDS buffer and detected by Western blot with anti-EZH2 antibody (Cell Signalling).

RNA In situ hybridization

Fluorescence labeled probe to detect *MEG3* was generated with a full length *MEG3* cDNA using the BioPrime Array CGH Genomic Labeling system (Invitrogen). BT-549 cells were grown in Culturewell™ MultiWell cell culture system (Molecular Probes) for 36 hours followed by fixation, probe hybridization, washing, image capturing and image processing were performed as in Reinius *et al.* [21].

Chromatin oligo affinity precipitation (ChOP)

Biotin labeled antisense DNA probes against full length *MEG3* were designed using online probe designer at http://www.singlemoleculefish.com. Probes showing non-*MEG3* homology with the human genome using BLAT were discarded. For the specific pull-down of *MEG3*, we have used 15 Biotin probes spanning whole *MEG3* transcript. A probe against GFP RNA was used as a negative control with no known target in human genome. *MEG3* ChOP was performed according to the protocol from Mariner *et al.* [22] with following modifications. Cross linking was performed with 1% glutaraldehyde and chromatin was sonicated to obtain chromatin fragment of 100-500 base pairs. Hybridization was carried out either with 100 pmol of *MEG3* probes (pooled 15 antisense biotin *MEG3* probes) or with 100 pmol control GFP probe at 37°C for 4 hr followed by capturing of the biotin probes with streptavidin-magnetic beads (Life technologies). For elution of the RNA and the associated DNA from the captured streptavidin-magnetic was performed as in Chu *et al.* [3]. *MEG3* enrichment in the eluted fraction was analyzed by RT-qPCR. The eluted DNA was subjected to library preparation and high-throughput sequencing using the Life Technologies SOLiD 5500xl instrumentation. The sequencing reads were aligned to the human reference genome (hg19) using LifeScope (v 2.5.0). All samples were sequenced in duplicates (technical duplicates)

and the total number of the aligned reads were; *MEG3* probes: 5,759,775 reads, GFP probe: 8,454,922 reads, and 21,615,528 reads for input. Analysis of the sequencing reads was basically carried out according to the pipeline as detailed in Chu *et al.* [3]. Peaks in the *MEG3* data were called using a peak calling software MACS (v 1.4.1.) [12] with both the GFP and input data as background. *MEG3* peaks were further filtered using the 'peak_correlation.pl' script (http://changlab.stanford.edu/chirpseq.tar.gz) requiring a Pearson correlation > 0.3 and a fold change enrichment over the combined GFP and input background > 1 between the replicates, resulting in 6837 *MEG3* peaks. For validation of the *MEG3* binding sites, ChOP was performed with both sense and antisense probes. Location of the sense probes was same as antisense probes but instead sense sequence of *MEG3* was used. For RNase A treated ChOP purification, chromatin was pretreated with 3 μL of RNase A (20 mg/mL, Life Technologies) at 37°C for 30 minutes prior to hybridization. Purified ChOP DNA was used in qPCR to check the enrichment of the selected *MEG3* peaks associated with the *TGF-β* genes using primers provided in the Supplementary Data 10.

Motif analysis and assignment of *MEG3* peaks to genes using GREAT tool

Sequences within ±200 bp around *MEG3* peak summits were extracted and motif analysis of these peaks was performed using MEME-Chip [23]. Motifs with the lowest *e*-value (most significant) were considered. *MEG3* peaks were assigned to genes with the help of GREAT tool using criteria of proximal distance 5 kb upstream, 2 kb downstream of transcription start sites and up to 500 kb distal to gene promoters [24].

Chromatin Immuno precipitation (ChIP)

H3K4me1 ChIP in BT-549 cells was performed with 3 μg of anti-H3K4me1 antibody (Santa Cruz) using protocol followed in Robertson *et al.* [25]. 100-300 ng of immunoprufied DNA from H3K4me1 ChIP was purified and sequenced on Illumina platform as reported in Robertson *et al.* [26]. Sequencing reads were analyzed and the enriched regions were defined by following the criteria from Robertson *et al.* [25]. Bedtools were used to calculate the closest distance between H3K4me1 peaks and the *MEG3* peaks [27].

EZH2 ChIP on CtrlSh and *MEG3*sh BT-549 cells was performed with 3 μg anti-EZH2 (Active motif) and anti-H3K27me3 (Millipore) using HighCell ChIP kit from Diagenode (A16) and following the protocol provided with the kit. The primers used in the qPCR reactions are provided in Supplementary Data 10.

Triplex-ChIP was carried out with anti dA.2rU antibody using ChIP protocol as in Kuo *et al.* [5] with following modification. BT-549 cells were fixed with 1% formaldehyde for 10 mins at RT. Nuclei were isolated from formaldehyde fixed cells using 1X nuclei isolation buffer (40mM Tris-HCl pH7.5, 20mM MgCl2, 4% tritonX-100, 1.28M sucrose). The nuclei were resuspended in lysis buffer (0.1%SDS, 0.5% TritonX 100, 20mM Tris-HCl pH7.5, 150mM NaCl) at a ratio of 1ml per 10 million of cells and sonicated (Bioruptor, around 30 cycles) to obtain chromatin fragments of about 500bp. Immunoprecipitation of chromatin with anti dA.2rU antibody [28] was carried out for 4-5 hours at 4°C. In case of pre-treatment with RNase A or RNase H chromatin was pre-treated with RNase A (2 µl of 20 mg/ml, Life Technologies) or with RNase H (15 units, NEB) for 30 minutes at 30°C. Incubation with Protein A magnetic beads, washing, chromatin elution from beads and ChIP DNA purification was carried out using protocol from Kuo *et al.* [5].

Functional analysis of the deregulated genes

Functional enrichment analysis of deregulated genes after *MEG3* and *EZH2* knockdown was done with the help of KEGG dataset using command line tool, Gene Set Clustering based on Functional annotation (GeneSCF). The PERL scripts from this tool are deposited in: https://github.com/santhilalsubhash/geneSCF. Fisher's exact test was used to obtain *P* values for enriched pathways and the documentation for statistics is presented along with the tool: https://github.com/santhilalsubhash/geneSCF/wiki/Statistical-methods-used. Pathway analysis was also performed on the genes which are commonly deregulated in Microarray and RNA-seq experiments.

Visualization of the pathways using Cytoscape

The functional networks of the 300 genes, which were deregulated after *MEG3* knockdown and were associated with at least one of the *MEG3* peaks, were constructed with the help of Cluepedia [29] on Cytoscape [30].

Analysis of *MEG3* expression in breast cancer subtypes

Expression levels of *MEG3* and target genes were assessed in a batch-corrected compendium of seventeen Affymetrix U133A/plus 2 primary breast tumors and three cell line gene expression datasets [31]. Briefly, Raw .cel files from seventeen Affymetrix U133A/plus 2 primary breast tumour and three cell line gene expression datasets were downloaded from

NCBI GEO (GSE12276, GSE21653, GSE3744, GSE5460, GSE2109, GSE1561, GSE17907, GSE2990, GSE7390, GSE11121, GSE16716, GSE2034, GSE1456, GSE6532, GSE3494), or caBIG (geral-00143) repositories, summarized with Ensembl alternative CDF [32] and normalized with RMA [33], before integrating using ComBat [34] to remove dataset-specific bias [35]. The intrinsic molecular subtypes were assigned based on the highest correlation to the Sorlie *et al.* [36] centroids for each subtype. To compare gene expression of *MEG3* in normal breast tissue and invasive ductal carcinoma the dataset GSE10780 was downloaded from NCBI GEO [37] and pre-processed as above using Ensembl aCDF and RMA normalization.

Nuclear and cytoplasmic RNA fractionation

BT-549 cells (1×10^6 cells) were resuspended in cold 175 µl RLN1 solution (50mM Tris HCl pH 8.0; 140mM NaCl; 1.5mM MgCl2; 0.5% NP-40; supplied with RNase inhibitor) and incubated 5 minutes on ice. Cell suspension was centrifuged at 4°C for 3 minutes at 300g. The supernatant, which corresponds to cytoplasmic fraction, was transferred into a new tube. The pellet, comprising nuclei, was resuspended in 175 µl of RLN2 solution (50mM Tris HCl pH 8.0; 500mM NaCl; 1.5mM MgCl2; 0.5% NP-40; supplied with RNase inhibitor) and incubated on ice for 5 minutes. The suspension was centrifuged at 4°C for 2 minutes at 16360g. The supernatant from this fraction corresponds to the nuclear soluble fraction. RNA was extracted from the cytoplasmic and nuclear fractions using Trizol. 1 µg of DNase I treated cytoplasmic and nuclear RNA was used for the RT-qPCR reaction.

Cell invasion assay

Invasion assay was performed using the biocoat invasion chamber (BD bioscience, 354480) following manufacturer's instructions. In brief, cells were seeded in 50,000-60,000 cells/ml density on the upper chamber in DMEM medium supplemented with 1% serum. DMEM medium containing 10 % serum was added to the lower chamber. The migrated cells were fixed and stained with SNABB-DIFF Kit (Labex AB, Sweden) and counted under light microscope.

Predicting Triplex Forming Oligo (TFO) and Triple-helix Target Site (TrTS)

Triplexator is a tool used in our analysis to predict the Triplex Forming Oligos (TFO's) and Triplex Target Sites (TrTS) [38]. The TFO's in *MEG3* transcript were considered using the default criteria by allowing 15 % error rate (number of mismatches) and less than 7 bp repeat length. To find TrTS over the *MEG3* peaks summit (±200 bp from centre of the peak, total

400bp) we have used default criteria set by Triplexator that is up to 50 % of Guanine content with an error rate of less than 10 % (number of mismatches). As a control, we have used the flanking sequences of the *MEG3* peak summit (200 bp up and 200 bp down-stream of the peak summit, total 400bp).

Electrophoretic mobility shift assay

Double stranded Oligonucleotides were end-labeled with T4 polynucleotide kinase in the presence of [γ-32P]ATP and purified using G-25 columns (GE Healthcare). To remove secondary structures present in *MEG3* TFO or control RNA, RNA oligonucleotides were heated at 70°C for 5 minutes followed by 5 minutes incubation on ice. Binding reaction was carried out in 10 µl of reaction volume and reaction mixtures contained (in order of addition) nuclease free water, labeled dsDNA oligonucleotides (0.4 pmol), 1 µl 10X Triplex forming buffer (100mM Tris pH 7.5, 250mM NaCl and 100mM MgCl2), 1 µl Yeast tRNA (1 mg/ml stock) followed by addition of 1.0 and 2.0 µM of RNA oligonucleotides and incubated for 2 hours at 25°C. For competition reactions, binding reaction were additionally supplied with 8 pmol (20 times more than labeled oligonucleotides) of non-labeled control or specific dsDNA oligonucleotides. Labeled dsDNA corresponding to *TGFBR1* associated *MEG3* peak sequence or a mutated version were incubated with increasing concentration (0.025, 0.5, 1.0, 2.0, or 4.0 µM) of *MEG3* ssRNA TFO. In control assay, triplex reaction was treated with either 5 units of RNase H (NEB) or 1µl of RNase A (20 mg/ml, Life Technologies) for 20min at 30°C. To crosscheck RNase H activity, we have digested an *in vitro* formed DNA-RNA hybrid for 20 min at 30°C. Triplex formation was monitored on 20 % polyacrylamide TBE gel (Life Technologies) in 1X TBE buffer supplemented with 8 mM MgCl2 at room temperature for 90 minutes at 200 volts. The details of the sequences used in the assay are provided in Supplementary Data 10.

Triplex capture assay

For *in vitro* and *in vivo* Triplex capture assay, we have followed a protocol by Besch *et al.* [39]. Nuclei from BT-549 cells were prepared by resuspending the cells in 1X nuclei isolation buffer (40mM Tris-HCl pH7.5, 20mM MgCl2, 4% tritonX-100, 1.28M sucrose) and incubation on ice for 20 minutes, followed by two washes with cold PBS. For *in vitro* triplex capture assay, 10 µM psoralen-biotinylated *MEG3* TFO or control TFO RNA oligonucleotides (purchased from Sigma-Aldrich) were incubated with the BT-549 nuclei (3×10^6 nuclei/ reaction) for 1 hour at 30°C in 100 µl of 1X Triplex forming buffer (10mM Tris pH 7.5,

25mM NaCl and 10mM MgCl2), followed by 5 minutes UV treatment to allow photoadduct formation. Nuclei were lysed by sonication using Bioruptor (10 cycles, 30 sec ON and 30sec OFF, Diagenode) followed by spin at 10,000 RPM to collect the supernatant. The Supernatants were incubated with 50µl streptavidin-magnetic beads (beads were pre-blocked with 2mg/mL BSA and 1mg/mL Yeast tRNA) at 37°C with rotation for 30 minutes. In case of RNase H control reaction, the supernatants were treated with 15 units of RNase H for 20 min at 30°C before streptavidin-magnetic beads were added. Following beads capture, the beads were washed 5 times in 1X Triplex forming buffer to remove the non-specifically bound DNA fragments and then beads were resuspended in 100 µl of DNA isolation buffer (50mM NaHCO3, 1% SDS, 200 mM NaCl). Resuspended beads bound to DNA-RNA triplex were treated with RNase A (2 µl, 20 mg/ml) for 30 minutes at 37°C followed by Proteinase K (Life Technologies) treatment at 55°C for 45 minutes. Subsequently 2- Mercaptoethanol (added up to final concentration of 2M, Sigma-Aldrich) and excess D-Biotin (added up to final conc of 2mM, Life Technologies) were mixed with the beads and heated at 95°C for 5 minutes to obtain the complete elution of the captured DNA from beads. DNA was purified with phenol-chloroform extraction and ethanol precipitation followed by qPCR.

For *in vivo* Triplex capture assay, 10 µM psoralen-biotinylated *MEG3* or control TFO were transfected into 1×10^6 BT-549 cells using Lipofectamine 2000 reagent (Life Technologies), and 36 hours post transfection, the cells were crosslinked with UV for 5 min. Nuclei isolation from the transfected and UV treated cells followed by nuclear lysis in 1X Triplex forming buffer by sonication and capturing of the biotinylated RNA oligo with streptavidin-magnetic beads was performed as described above. As a control, UV cross-linked lysates from *in vivo* Triplex capture assays were treated with 15 units RNase H for 20min at 30°C before incubation with streptavidin-magnetic beads. DNA bound to beads was eluted and purified using the same method described in *in vitro* Triplex capture assay. To check the chromatin interacting property of WT and mutant *MEG3* RNAs (**Δ** 345-348 and **Δ** 46-56 *MEG3*), we followed *in vivo* Triplex capture assay procedure. In this assay, instead of psoralen-biotin RNA TFO, 2 µg of biotin labeled WT and mutant RNAs were transfected into BT-549 cells using Lipofectamine 2000 reagent (Life Technologies). Biotin labeled WT and mutant *MEG3* RNAs were *in vitro* transcribed with T7 RNA polymerase (Promega) using biotin RNA labeling Mix (Roche) and RNA quality was assessed in 0.8% agarose gel. 36 hours post RNA transfection, formaldehyde (1%) crosslinking was performed followed by nuclei isolation,

nuclei lysis, chromatin fragmentation by sonication, streptavidin-magnetic bead pulldown and DNA isolation and purification following the same protocol described above.

CD spectroscopy

CD-spectra were recorded on a Jasco J-810 spectropolarimeter. Each spectrum is the average of 2-3 consecutive spectra (technical replicate) measured 5s per datapoint, and baseline-corrected with a spectrum of pure buffer. CD is defined as the difference in absorbance of left and right circularly polarized light: $CD(\lambda)=A_l(\lambda)-A_r(\lambda)$. CD-spectra were recorded on *MEG3* ssRNA TFO (2.2 μM) and the three different dsDNA oligos (corresponding to the *TGFB2, TGFBR1*, and *SMAD2* genes associated *MEG3* peaks, 2.2 μM each) separately as well as on a 1:1 mix of the two (2.2 μM ssRNA and 2.2 μM dsDNA oligo) in 1X triplex forming buffer (10mM Tris pH 7.5, 25mM NaCl and 10mM MgCl2). For comparison, sample with a control ssRNA was included (negative control). The mixed samples or individual RNA and dsDNAs were equilibrated approximately 1h at 30°C. The measurements were performed at room temperature in a 1 mm cuvette using 200 μL solutions and kept at room temperature before the measurement. The data presented in the spectra is the Molar Ellipticity given based on the concentration of nucleotides in the sample.

Western Blot analysis

Cells were harvested 48 hours post-siRNA transfection and were lysed in lysis buffer [1%Triton X-100, 150 mM Nacl , 10 mM Tris HCL pH 7.4 , 1 m*M* EGTA , 1 m*M* EDTA, 0,5% NP-40 with protease inhibitors cocktail]. 10 μg of each cell lysate was ran on NuPAGE 4-12% Bis-Tris gel (Novex Invitrogen, San Diego, CA). Proteins were transferred to a nitrocellulose membrane (Hybond ECL, GE healthcare). The membrane was blocked with blocking buffer (5% bovine serum albumin (BSA) in 1x TBS-T (10 mM Tris-base, 0.15 M NaCl, Ph 7.7 and 2% Tween) for 1 hour at room temperature. Blocked membrane was incubated overnight at 4°C with primary antibodies anti-EZH2 (1:1000 dilution, Cell Signalling), Anti-Tubulin (1:1000 dilution, Millipore), TGFBRI (1:500 dilution, Santa Cruz Biotechnology), SMAD2 (1:1000 dilution, home-made rabbit anti serum from Aristidis Moustakas laboratory), followed by 1 hour incubation with secondary antibody Goat Anti-Mouse IgG, 1:1000 dilution (Milipore), and Goat Anti-Rabbit IgG, 1:1000 dilution (Milipore) at room temperature. Blots were developed in substrate solution (Chemoluminiscence Super Signal, Pierce Chemical Co) and were visualized using IMAGE BioRad 4.0 alpha software

(BioRad gel imaging system). Uncropped scans of the blots are provided in the supplementary information (Supplementary Fig. 13).

Chromatin conformation capture (3C)

3C was performed according to the published protocol [40]. In brief, 2% formaldehyde fixed chromatin from Ctrlsh or *MEG3*sh BT-549 cells was digested with EcoRI. After ligation and DNA purification, we analyzed the 3C interactions between the *TGFBR1* associated distal *MEG3* peak and the *TGFBR1* promoter in Ctrlsh or *MEG3*sh BT-549 cells using the primers provided in Supplementary Data 10. In order to measure the 3C interactions accurately, the difference in efficiency between the primers was normalized with a control BAC clone covering the whole *TGFBR1* locus. The BAC clone was digested with EcoRI followed by ligation to generate all possible random combinations of ligation products. This ligated DNA was then used to check the efficiencies of the different primer pairs. 3C-qPCR data was normalized with the control 3C primers to eliminate the minor difference in cross-linking between the cell lines and also with the *GAPDH* primers for input amount (loading control).

Luciferase Assay

A 1.4 kb region corresponding to *MEG3* peak upstream of the *TGFBR1* gene was PCR amplified with primer as mentioned in Supplementary Data 10 and cloned into BamHI and SalI sites of pGL3 promoter vector (Promega) containing the reporter firefly luciferase gene. 70,000 Ctrlsh and *MEG3*sh BT-549 cells were plated in 24 well and transfected with 200 ng of pGL3 promoter vector containing the 1.4 kb fragment along with 200 ng of *Renilla* Luciferase vector (Promega) using lipofectamine 2000 reagent (Life Technologies). Luminescence was measured 48 hours post-transfection using Dual-Glo Luciferase Assay System (Promega). Expression of firefly luciferase was normalized with *Renilla* luciferase and data was represented as relative expression compared to the Ctrlsh cells.

Immunofluorescence

BT-549 cells were plated on glass coverslips at densities of 50,000 to 60,000 cells/ml and cells were allowed to attach for 24 hours. The cells were fixed with 3.7% formaldehyde (pH 7.4) in PBS for 15 min followed by 3X PBS wash. The cells were permeabilized in PBS containing 0.25% Triton X-100 (PBST) for 10 minutes, followed by 3X PBS wash. Following permeabilization, the cells were treated with RNase A or with RNase H at 30°C for 20 minutes. Control cells were incubated at 30°C for 20 mins in PBS without any treatment.

Blocking was performed with 1% Bovine Serum Albumin (BSA) in PBST for 30 minutes. Permeabilized and BSA blocked BT-549 cells were incubated with anti dA.2rU antibody [28] (1:50 dilution in PBST) for 1hour at room temperature followed by three washes with 1X PBS. Incubation with secondary antibody (fluorescent-labeled, AlexaFluor488) was carried in the dark for one hour followed by three PBS washes. The cover slips were mounted on a slide using Vectashield (Vector laboratories) containing DAPI. For the labeling of mitochondria MitoTracker Red CMXRos (lifetechnologies) was added to the culture media and incubated for 30 minutes followed by fixation and immuno staining following the same protocol described above. Fluorescent microscopic images were obtained with Leica DFC 340FX microscope. 3D-images were acquired using an inverted LSM 700 confocal microscope (Carl Zeiss, Germany) using a Plan-Apochromat 63x/1.40 oil objective and a pinhole setting of 1 AU. DAPI was excited at 405 nm and emission was detected between 400-492 nm, AlexaFluor488 was excited at 488 nm and the emission detected between 400-530 nm, when red fluorophore was used and or 492-700 nm when only with DAPI. MitoTracker Red CMXRos Orange was excited at 555 nm and the emission was detected at 560-700 nm. The channels were acquired sequentially to avoid bleed though. Segmentation and signal intensities were measured in Volocity (PerkinElmer Inc). The total AlexaFluor 488 signal intensity in the nucleus or cytoplasm was normalized by the volume of the areas, respectively.

Immunodot

Nucleic acid solutions, previously quantified by spectrophotometry (Cary 50 Bio UV, Varian), were dotted onto nitrocellulose membranes (Bio-Rad). After air drying, nucleic acids (3 µg per dot) were UV-fixed (GS Genelinker, Bio-Rad) and the strips incubated overnight at room temperature, on an orbital shaker, in 2X SSC, 0.1%SDS pH 5.5 containing either nucleic acid homopolymers or oligo-deoxyribonucleotides (200 ng/ml). After washings in 2X SSC, 0.2% SDS at room temperature, the strips were left at room temperature in 1X TBS, 0.1% Triton X-100 (TBST) pH 7.4, 2% low fat powdered milk for 30 minutes. Anti-(dA)/2(rU) was diluted 1:500 in the above solution and the incubation was done at room temperature for 2 hours on an orbital shaker. The strips were washed twice in TBST for 10 minutes and then incubated in TBST solution containing goat anti-rabbit IgG conjugated with alkaline phosphatase (Sigma) diluted 1:3000 for 1 hour at room temperature. The strips were then washed twice in TBST for 10 minutes and finally in 1X TBS for 5 minutes. Color development was performed with BCIP/NBT phosphatase substrate according to the manufacturer's instructions (KPL). Single and double-stranded homopolymers were

purchased from Roche and GE Healthcare; (dGAA)7.(dCTT)7 duplex was made by mixing equimolar amounts of complementary single-stranded sequences (Life Tech.) in 2X SSC. The mixture was heated at 85ºC and allowed to cool at room temperature for 1 hour.

Supplementary References

1. Zuo, T. et al. Epigenetic silencing mediated through activated PI3K/AKT signaling in breast cancer. *Cancer Res* **71**, 1752-62 (2011).
2. Lam, A.L., Boivin, C.D., Bonney, C.F., Rudd, M.K. & Sullivan, B.A. Human centromeric chromatin is a dynamic chromosomal domain that can spread over noncentromeric DNA. *Proc Natl Acad Sci U S A* **103**, 4186-91 (2006).
3. Chu, C., Qu, K., Zhong, F.L., Artandi, S.E. & Chang, H.Y. Genomic maps of long noncoding RNA occupancy reveal principles of RNA-chromatin interactions. *Mol Cell* **44**, 667-78 (2011).
4. Mondal, T., Rasmussen, M., Pandey, G.K., Isaksson, A. & Kanduri, C. Characterization of the RNA content of chromatin. *Genome Res* **20**, 899-907 (2010).
5. Kuo, M.H. & Allis, C.D. In vivo cross-linking and immunoprecipitation for studying dynamic Protein:DNA associations in a chromatin environment. *Methods* **19**, 425-33 (1999).
6. Li, H. et al. The Sequence Alignment/Map format and SAMtools. *Bioinformatics* **25**, 2078-9 (2009).
7. Trapnell, C. et al. Transcript assembly and quantification by RNA-Seq reveals unannotated transcripts and isoform switching during cell differentiation. *Nat Biotechnol* **28**, 511-5 (2010).
8. Harrow, J. et al. GENCODE: the reference human genome annotation for The ENCODE Project. *Genome Res* **22**, 1760-74 (2012).
9. Roberts, A., Pimentel, H., Trapnell, C. & Pachter, L. Identification of novel transcripts in annotated genomes using RNA-Seq. *Bioinformatics* **27**, 2325-9 (2011).
10. Robinson, M.D., McCarthy, D.J. & Smyth, G.K. edgeR: a Bioconductor package for differential expression analysis of digital gene expression data. *Bioinformatics* **26**, 139-40 (2010).
11. Plaisier, S.B., Taschereau, R., Wong, J.A. & Graeber, T.G. Rank-rank hypergeometric overlap: identification of statistically significant overlap between gene-expression signatures. *Nucleic Acids Res* **38**, e169 (2010).
12. Zhang, Y. et al. Model-based analysis of ChIP-Seq (MACS). *Genome Biol* **9**, R137 (2008).
13. Pollard, K.S., Hubisz, M.J., Rosenbloom, K.R. & Siepel, A. Detection of nonneutral substitution rates on mammalian phylogenies. *Genome Res* **20**, 110-21 (2010).
14. Wang, L. et al. CPAT: Coding-Potential Assessment Tool using an alignment-free logistic regression model. *Nucleic Acids Res* **41**, e74 (2013).
15. Genomes Project, C. et al. An integrated map of genetic variation from 1,092 human genomes. *Nature* **491**, 56-65 (2012).
16. Kaneko, S. et al. Interactions between JARID2 and noncoding RNAs regulate PRC2 recruitment to chromatin. *Mol Cell* **53**, 290-300 (2014).
17. International Human Genome Sequencing, C. Finishing the euchromatic sequence of the human genome. *Nature* **431**, 931-45 (2004).
18. Li, R. et al. SOAP2: an improved ultrafast tool for short read alignment. *Bioinformatics* **25**, 1966-7 (2009).

19. Guttman, M. et al. lincRNAs act in the circuitry controlling pluripotency and differentiation. *Nature* **477**, 295-300 (2011).
20. Tsai, M.C. et al. Long noncoding RNA as modular scaffold of histone modification complexes. *Science* **329**, 689-93 (2010).
21. Reinius, B. et al. Female-biased expression of long non-coding RNAs in domains that escape X-inactivation in mouse. *BMC Genomics* **11**, 614 (2010).
22. Mariner, P.D. et al. Human Alu RNA is a modular transacting repressor of mRNA transcription during heat shock. *Mol Cell* **29**, 499-509 (2008).
23. Machanick, P. & Bailey, T.L. MEME-ChIP: motif analysis of large DNA datasets. *Bioinformatics* **27**, 1696-7 (2011).
24. McLean, C.Y. et al. GREAT improves functional interpretation of cis-regulatory regions. *Nat Biotechnol* **28**, 495-501 (2010).
25. Robertson, A.G. et al. Genome-wide relationship between histone H3 lysine 4 mono- and tri-methylation and transcription factor binding. *Genome Res* **18**, 1906-17 (2008).
26. Robertson, G. et al. Genome-wide profiles of STAT1 DNA association using chromatin immunoprecipitation and massively parallel sequencing. *Nat Methods* **4**, 651-7 (2007).
27. Quinlan, A.R. & Hall, I.M. BEDTools: a flexible suite of utilities for comparing genomic features. *Bioinformatics* **26**, 841-2 (2010).
28. Gorab, E., Amabis, J.M., Stocker, A.J., Drummond, L. & Stollar, B.D. Potential sites of triple-helical nucleic acid formation in chromosomes of Rhynchosciara (Diptera: Sciaridae) and Drosophila melanogaster. *Chromosome Res* **17**, 821-32 (2009).
29. Bindea, G., Galon, J. & Mlecnik, B. CluePedia Cytoscape plugin: pathway insights using integrated experimental and in silico data. *Bioinformatics* **29**, 661-3 (2013).
30. Smoot, M.E., Ono, K., Ruscheinski, J., Wang, P.L. & Ideker, T. Cytoscape 2.8: new features for data integration and network visualization. *Bioinformatics* **27**, 431-2 (2011).
31. Moleirinho, S. et al. KIBRA exhibits MST-independent functional regulation of the Hippo signaling pathway in mammals. *Oncogene* **32**, 1821-30 (2013).
32. Dai, M. et al. Evolving gene/transcript definitions significantly alter the interpretation of GeneChip data. *Nucleic Acids Res* **33**, e175 (2005).
33. Irizarry, R.A. et al. Exploration, normalization, and summaries of high density oligonucleotide array probe level data. *Biostatistics* **4**, 249-64 (2003).
34. Johnson, W.E., Li, C. & Rabinovic, A. Adjusting batch effects in microarray expression data using empirical Bayes methods. *Biostatistics* **8**, 118-27 (2007).
35. Sims, A.H. et al. The removal of multiplicative, systematic bias allows integration of breast cancer gene expression datasets - improving meta-analysis and prediction of prognosis. *BMC Med Genomics* **1**, 42 (2008).
36. Sorlie, T. et al. Repeated observation of breast tumor subtypes in independent gene expression data sets. *Proc Natl Acad Sci U S A* **100**, 8418-23 (2003).
37. Chen, D.T. et al. Proliferative genes dominate malignancy-risk gene signature in histologically-normal breast tissue. *Breast Cancer Res Treat* **119**, 335-46 (2010).
38. Buske, F.A., Bauer, D.C., Mattick, J.S. & Bailey, T.L. Triplexator: detecting nucleic acid triple helices in genomic and transcriptomic data. *Genome Res* **22**, 1372-81 (2012).

39. Besch, R., Giovannangeli, C., Schuh, T., Kammerbauer, C. & Degitz, K. Characterization and quantification of triple helix formation in chromosomal DNA. *J Mol Biol* **341**, 979-89 (2004).
40. Hagege, H. et al. Quantitative analysis of chromosome conformation capture assays (3C-qPCR). *Nat Protoc* **2**, 1722-33 (2007).
